# Loss of Prohibitin Membrane Scaffolds Impairs Mitochondrial Architecture and Leads to Tau Hyperphosphorylation and Neurodegeneration

**DOI:** 10.1371/journal.pgen.1003021

**Published:** 2012-11-08

**Authors:** Carsten Merkwirth, Paola Martinelli, Anne Korwitz, Michela Morbin, Hella S. Brönneke, Sabine D. Jordan, Elena I. Rugarli, Thomas Langer

**Affiliations:** 1 Institute for Genetics, University of Cologne, Cologne, Germany; 2 Cologne Excellence Cluster on Cellular Stress Responses in Aging-Associated Diseases (CECAD), University of Cologne, Cologne, Germany; 3 Center for Molecular Medicine (CMMC), University of Cologne, Cologne, Germany; 4 Institute for Zoology, University of Cologne, Cologne, Germany; 5 Neuropathology and Neurology 5, IRCCS Foundation, Neurological Institute Carlo Besta, Milano, Italy; 6 Max Planck Institute for Biology of Aging, Cologne, Germany; Max Planck Institute for Biology of Aging, Germany

## Abstract

Fusion and fission of mitochondria maintain the functional integrity of mitochondria and protect against neurodegeneration, but how mitochondrial dysfunctions trigger neuronal loss remains ill-defined. Prohibitins form large ring complexes in the inner membrane that are composed of PHB1 and PHB2 subunits and are thought to function as membrane scaffolds. In *Caenorhabditis elegans,* prohibitin genes affect aging by moderating fat metabolism and energy production. Knockdown experiments in mammalian cells link the function of prohibitins to membrane fusion, as they were found to stabilize the dynamin-like GTPase OPA1 (optic atrophy 1), which mediates mitochondrial inner membrane fusion and cristae morphogenesis. Mutations in OPA1 are associated with dominant optic atrophy characterized by the progressive loss of retinal ganglion cells, highlighting the importance of OPA1 function in neurons. Here, we show that neuron-specific inactivation of *Phb2* in the mouse forebrain causes extensive neurodegeneration associated with behavioral impairments and cognitive deficiencies. We observe early onset tau hyperphosphorylation and filament formation in the hippocampus, demonstrating a direct link between mitochondrial defects and tau pathology. Loss of PHB2 impairs the stability of OPA1, affects mitochondrial ultrastructure, and induces the perinuclear clustering of mitochondria in hippocampal neurons. A destabilization of the mitochondrial genome and respiratory deficiencies manifest in aged neurons only, while the appearance of mitochondrial morphology defects correlates with tau hyperphosphorylation in the absence of PHB2. These results establish an essential role of prohibitin complexes for neuronal survival *in vivo* and demonstrate that OPA1 stability, mitochondrial fusion, and the maintenance of the mitochondrial genome in neurons depend on these scaffolding proteins. Moreover, our findings establish prohibitin-deficient mice as a novel genetic model for tau pathologies caused by a dysfunction of mitochondria and raise the possibility that tau pathologies are associated with other neurodegenerative disorders caused by deficiencies in mitochondrial dynamics.

## Author Summary

Mitochondria are the major site of cellular ATP production and are essential for the survival of neurons. High ATP levels are required to sustain neuronal activities and axonal transport of macromolecules and organelles. The functional integrity of mitochondria depends on fusion and fission of their membranes, which maintain a dynamic mitochondrial network in cells. Interference with these processes causes neurodegenerative disorders that are characterized by axonal degeneration of distinct neurons. However, how an impaired fusion affects mitochondrial activities and neuronal survival remains poorly understood. Here, we have addressed this question by analyzing forebrain-specific knockout mice lacking prohibitins. Prohibitin complexes form membrane scaffolds in the inner membrane, which we now show are required for mitochondrial fusion, ultrastructure, and genome stability in neurons. Loss of prohibitins triggers extensive neurodegeneration associated with behavioral and cognitive deficiencies. Surprisingly, we observe hyperphosphorylation and filament formation of the microtubule-associated protein tau, reminiscent of a large group of neurodegenerative disorders termed tauopathies. Our findings, therefore, not only provide new insight into how defects in mitochondrial fusion affect neuronal survival, but also point to an intimate relationship of deficiencies in mitochondrial dynamics and tau pathologies.

## Introduction

The dynamic behavior of mitochondria that constantly divide and fuse is pivotal to maintain their pleiotropic activities and their distribution within cells. Conserved protein machineries in the outer and inner membrane of mitochondria mediate membrane fusion events, ensure cristae formation and regulate the interaction of mitochondria with the endoplasmic reticulum [1]–[3]. Loss of mitochondrial fusion leads to neuronal loss in mice, highlighting the vulnerability of neurons for deficiencies in mitochondrial dynamics [4]–[6]. Mutations in the dynamin-like GTPases MFN2 and OPA1, which mediate mitochondrial membrane fusion, cause neurodegeneration in Charcot-Marie-Tooth disease type 2A and autosomal dominant optic atrophy, respectively [7]–[9]. Moreover, defects in mitochondrial dynamics are associated with multiple neurodegenerative diseases, including Parkinson's, Alzheimer's (AD) and Huntington's disease [10]–[12].

Recent evidence identified prohibitins in the mitochondrial inner membrane as novel modulators of mitochondrial fusion [13]–[15]. Prohibitins comprise a conserved and ubiquitously expressed protein family [16], [17]. Two homologous proteins, prohibitin-1 (PHB1) and prohibitin-2 (PHB2), assemble into large ring complexes in the inner membrane with putative functions as protein and lipid scaffolds [18]. The genetic interaction of yeast *PHB1* and *PHB2* with genes involved in the mitochondrial cardiolipin and phosphatidyl ethanolamine metabolism suggests that prohibitin complexes may also affect the lipid distribution in the inner membrane [19]. Consistently, PHB1 and PHB2 are homologous to members of the SFPH-family that were found in association with membrane microdomains in various cellular membranes [20], [21].

Despite emerging evidence for a scaffold function of prohibitins [16], only limited information is available on the physiological relevance of a defined spatial organization of the inner membrane for mitochondrial activities. Loss of prohibitin genes in *Caenorhabditis elegans* and mice results in embryonic lethality, pointing to essential functions during embryonic development [22], [23]. Knockdown of PHB1 and PHB2 in adult, non-neuronal tissues of *C. elegans* influences aging by moderating fat metabolism and energy production [24]. However, it remained unclear whether prohibitins affect mitochondrial respiratory activities directly. In mammalian cells, prohibitins appear to affect mitochondrial respiration in a cell-type specific manner. While knockdown of PHB1 impaired complex I activity in endothelial cells [25], mitochondrial respiratory function was not affected in prohibitin-deficient mouse embryonic fibroblasts (MEFs) [13]. These studies identified the processing of OPA1 as the central process regulated by prohibitins *in vitro*. The function of OPA1 in mitochondrial fusion and cristae morphogenesis depends on the presence of both long and short forms of OPA1, the latter being generated by proteolytic processing of long forms [26]–[29]. Loss of PHB2 destabilizes long OPA1 forms and inhibits mitochondrial fusion, resulting in the fragmentation of the mitochondrial network and an increased susceptibility of the cells towards apoptotic stimuli [13], [15]. Interestingly, a destabilization of long OPA1 forms has also been observed in cells lacking *m*-AAA proteases [30], ATP-dependent quality control enzymes with regulatory functions during mitochondrial biogenesis [4], which assemble with prohibitin complexes in the inner membrane of yeast, mammalian and plant mitochondria [31], [32]. Mutations in *m*-AAA protease subunits cause axonal degeneration in spinocerebellar ataxia, hereditary spastic paraplegia, and a spastic-ataxia neuropathy syndrome [33]–[35].

These results prompted us to assess *in vivo* the role of prohibitins in neurons, which contain high levels of prohibitins and are particularly vulnerable to disturbances in mitochondrial dynamics. Using conditional gene ablation in mice, we demonstrate that a post-natal loss of PHB2 in the forebrain triggers massive neurodegeneration which is associated with the accumulation of aberrant mitochondria and hyperphosphorylation of the microtubule-associated protein tau.

## Results

### Forebrain-specific PHB2-deficient mice

**Figure 1:**
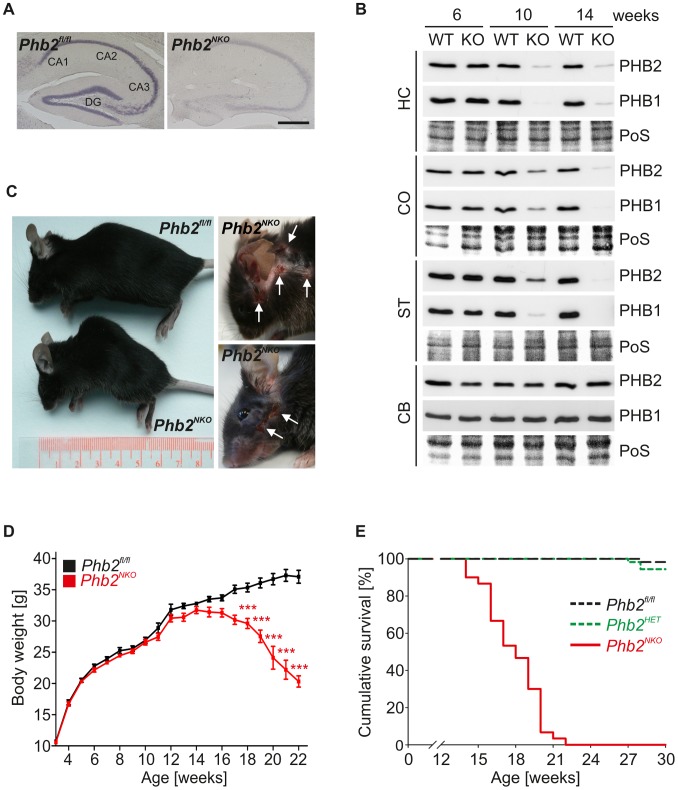
*CaMKIIa-Cre*-mediated inactivation of the mouse *Phb2* gene in forebrain neurons. (A) *In-situ* hybridization of *Phb2* mRNA in the hippocampus of 8-week-old *Phb2^NKO^* and *Phb2^fl/fl^* control mice. Scale bar: 500 µm. (B) Immunoblot analysis of tissue lysates generated from the indicated brain regions of *Phb2^NKO^* (KO) and *Phb2^fl/fl^* (WT) control mice of different age using PHB1- and PHB2-specific antibodies. Ponceau S (PoS) staining was used to monitor equal gel loading. Cortex (CO), striatum (ST), hippocampus (HC), cerebellum (CB). (C) Representative photographs of 20-week-old *Phb2^NKO^* mice of the indicated genotypes showing lordokyphosis (left panel) and excessive pathological grooming (right panel). White arrows indicate regions of self-inflicted open skin lesions. (D) Body weight analysis of *Phb2^NKO^* and *Phb2^fl/fl^* control animals. n = 20. ***P<0.001. Error bars indicate SEM. (E) Kaplan-Meier survival plot of *Phb2^NKO^* (n = 30) and control animals (*Phb2^fl/fl^* (n = 59), *Phb2^HET^* (n = 19)). *P*<0.0001.

Previous experiments using a genetic *loss-of-function* approach to uncover physiological functions of PHB2 revealed an early embryonic lethality phenotype in mice [13], [23]. To circumvent gene ablation during embryogenesis, conditional *Phb2* mice (*Phb2^fl/fl^*) were bred to mice expressing the Cre recombinase under control of the postnatally expressed *CaMKIIα* promoter (*CaMKIIα-Cre*) [36] resulting in neuron-specific PHB2-deficient mice (*Phb2^fl/fl;CaMKIIα-Cre^*; hereafter referred to as *Phb2^NKO^* mice). This mouse line shows a defined and restricted recombination pattern and a progressive increase in recombination efficiency after completed neuronal development [36]. Histological examinations of brains derived from *CaMKIIα-Cre* mice crossed to *ROSA26-LacZ* reporter mice revealed selective Cre-mediated recombination in forebrain regions including the cortex, striatum and hippocampus, to a minor extent in hypothalamic regions, but not in hind- and midbrain regions like the cerebellum (Figure S1) [37]. To demonstrate efficient depletion of *Phb2*, *in-situ* hybridization against the endogenous *Phb2* mRNA was performed. Notably, *Phb2* mRNA was virtually depleted in hippocampal neurons of 8-week-old *Phb2^NKO^* mice (Figure 1A). Consistently, immunoblotting of tissue lysates prepared from various brain compartments of mice of different age revealed maximal depletion of PHB2 in Cre-expressing tissues at 14-weeks, but not in the cerebellum where Cre recombinase is not expressed (Figure 1B). Notably, PHB2 depletion was accompanied by efficient loss of its assembly partner PHB1 (Figure 1B). This observation is consistent with previous findings in cultured MEFs [13] and demonstrates that prohibitin subunits are functionally interdependent in neurons *in vivo*.

Homozygous *Phb2^NKO^* mice were born at expected mendelian ratios, showed normal fertility and were anatomically indistinguishable from their WT littermates. From 12 to 14 weeks of age, however, *Phb2^NKO^* mice progressively developed aging-related phenotypes, including weight loss, cachexia and kyphosis (Figure 1C, 1D; Figure S2). Furthermore, *Phb2^NKO^* mice, but not control littermates, showed an excessive pathological grooming behavior characterized by facial hair loss and self-inflicted facial lesions (Figure 1C). An extensive analysis of behavioral and cognitive abilities in early-stage 8-week-old *Phb2^NKO^* animals revealed decreased hippocampus-dependent learning abilities and memory formation (Figure S3), and an impairment of innate fear behavior and motor coordination (Figure S4) (for details, see Text S1). The phenotypes of *Phb2^NKO^* animals deteriorated with age and led to premature death of *Phb2^NKO^* mice starting at the age of 14 weeks (Figure 1E). The maximal lifespan of *Phb2^NKO^* mice was 22 weeks only. Survival was not affected in homozygous *Phb2^fl/fl^* or heterozygous *Phb2^fl/WT;CaMKIIα-Cre^* (*Phb2^HET^*) mice (Figure 1E). We therefore conclude that PHB2 in the forebrain is essential for postnatal mouse survival.

### Progressive forebrain atrophy and neuronal loss in *Phb2^NKO^* mice

**Figure 2:**
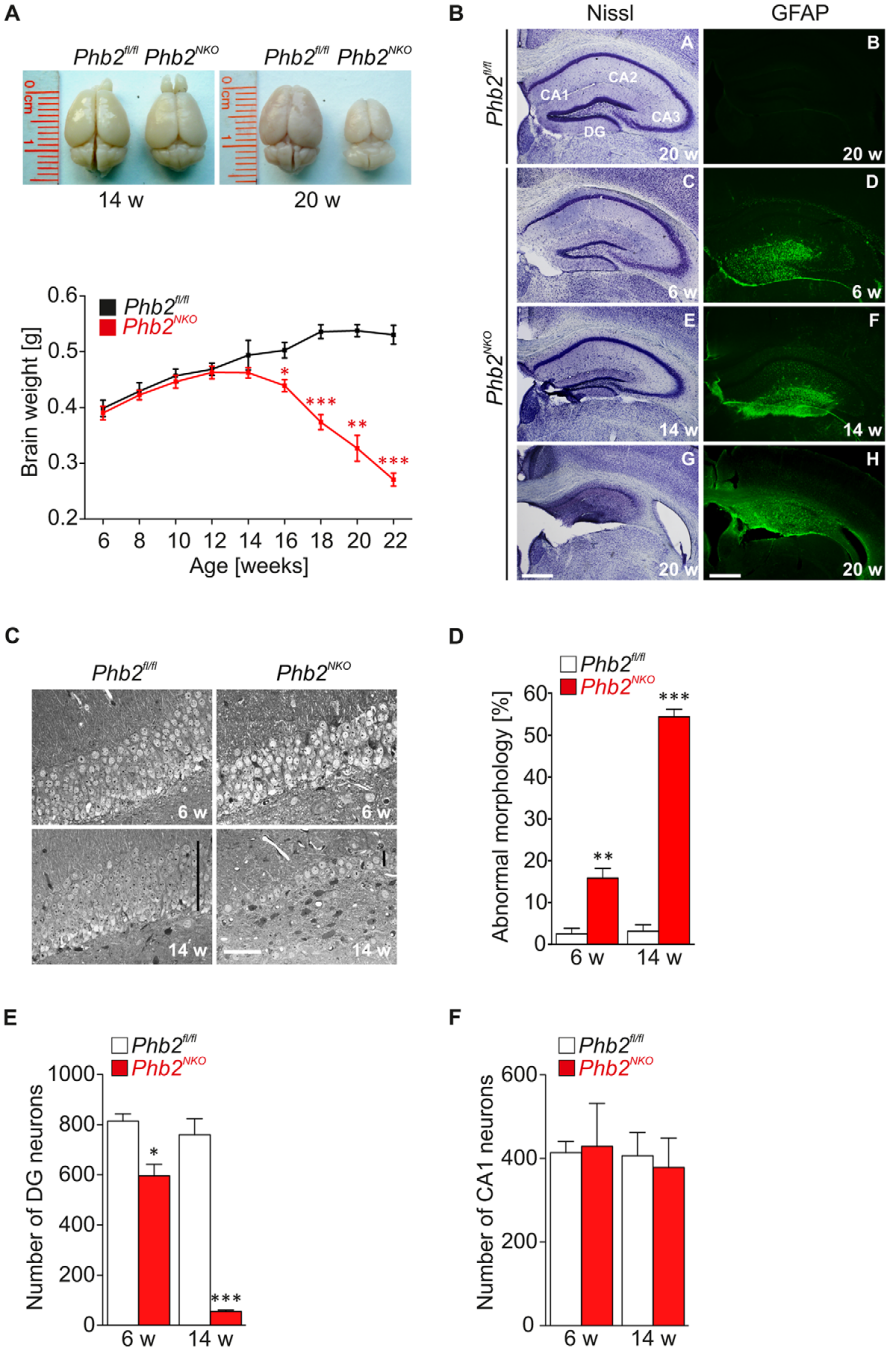
Progressive astrogliosis and loss of hippocampal neurons in *Phb2^NKO^* mice. (A) Representative photographs of brains isolated from 14- or 20-week-old *Phb2^NKO^* and *Phb2^fl/fl^* control mice (upper panel). Brain weights of *Phb2^NKO^* and *Phb2^fl/fl^* control animals were monitored at the indicated time points (lower panel). n = 5 per genotype and time point, *P<0.05; **P<0.01; ***P<0.001. Error bars indicate SEM. (B) Nissl staining of coronal sections across the hippocampal region from *Phb2^NKO^* and *Phb2^fl/fl^* control brains of the indicated age (left panel). Immunohistochemistry using GFAP antibody reveals progressive astrogliosis in the hippocampus of *Phb2^NKO^* mice (right panel). Scale bars: 400 µm. (C) Coronal semithin sections of the hippocampal DG from 6-week (upper panel) or 14-week-old (lower panel) *Phb2^NKO^* and *Phb2^fl/fl^* control mice. Black vertical bars show the thickness of the neuronal layers. White scale bar: 40 µm. (D) Quantification of neurons with degenerative features and vacuolization in the DG of *Phb2^NKO^* and *Phb2^fl/fl^* control mice of the indicated age. Data are expressed as percentage of total cells counted. At least 200 cells were scored per section; in case of the *Phb2^NKO^* at 14 weeks all residual neurons were scored. Error bars indicate SEM (n = 3). (E) Number of DG and (F) CA1 neurons in 6- and 14-week-old *Phb2^NKO^* mice. Error bars indicate SEM (n = 3).

To investigate the underlying defects at the cellular level, we analyzed gross brain morphology of *Phb2^NKO^* and control brains. *Phb2^NKO^* brains were indistinguishable from controls in size, weight and gross morphology at 14 weeks of age (Figure 2A). In contrast, at the age of 20 weeks we observed a massive atrophy of *Phb2^NKO^* forebrains, which was accompanied by a severe total brain weight loss (Figure 2A). Histological examinations of *Phb2^NKO^* brains further supported the progressive nature and severity of the phenotypes. Nissl stainings and semithin sections from *Phb2^NKO^* animals revealed that the region most prominently affected was the hippocampus, which undergoes progressive degeneration over time, culminating in the almost complete loss of neurons in both the dentate gyrus (DG) and cornu ammonis (CA) regions at 20 weeks of age (Figure 2B, Figure S5A). At this age, cortical neurons in all layers also appeared affected in *Phb2^NKO^* mice, showing shrinkage of the cell body and loss of processes (Figure S5B). Since the hippocampal region appeared to be a preferential target in the absence of PHB2, we analyzed this area in more detail. Neuronal loss was accompanied by a progressive development of astrogliosis, as demonstrated by increased GFAP reactivity already observable at 6 weeks of age in the DG (Figure 2B). At this age, a significant fraction of DG neurons in *Phb2^NKO^* mice appeared vacuolated and neuronal loss was already apparent (Figure 2D, 2E). At 14 weeks, the DG consisted of only one neuronal layer, with more than 50% of residual neurons showing degenerative features (Figure 2C–2E). Remarkably, while DG neurons were markedly reduced in number already at 14 weeks (Figure 2E), neurons in the CA1 region were less affected and neuronal loss became apparent only in 20-week-old *Phb2^NKO^* mice (Figure 2F, Figure S6). TUNEL staining of the hippocampal DG regions revealed few positive neuronal cell bodies, suggesting that neuronal loss in *Phb2^NKO^* brains is at least partially caused by apoptosis (Figure S5). We therefore conclude that PHB2 is generally required for neuronal survival *in vivo*. However, the time-course and severity of neuronal degeneration show regional differences.

### Loss of prohibitins affects the structural integrity and distribution of mitochondria in neurons

**Figure 3:**
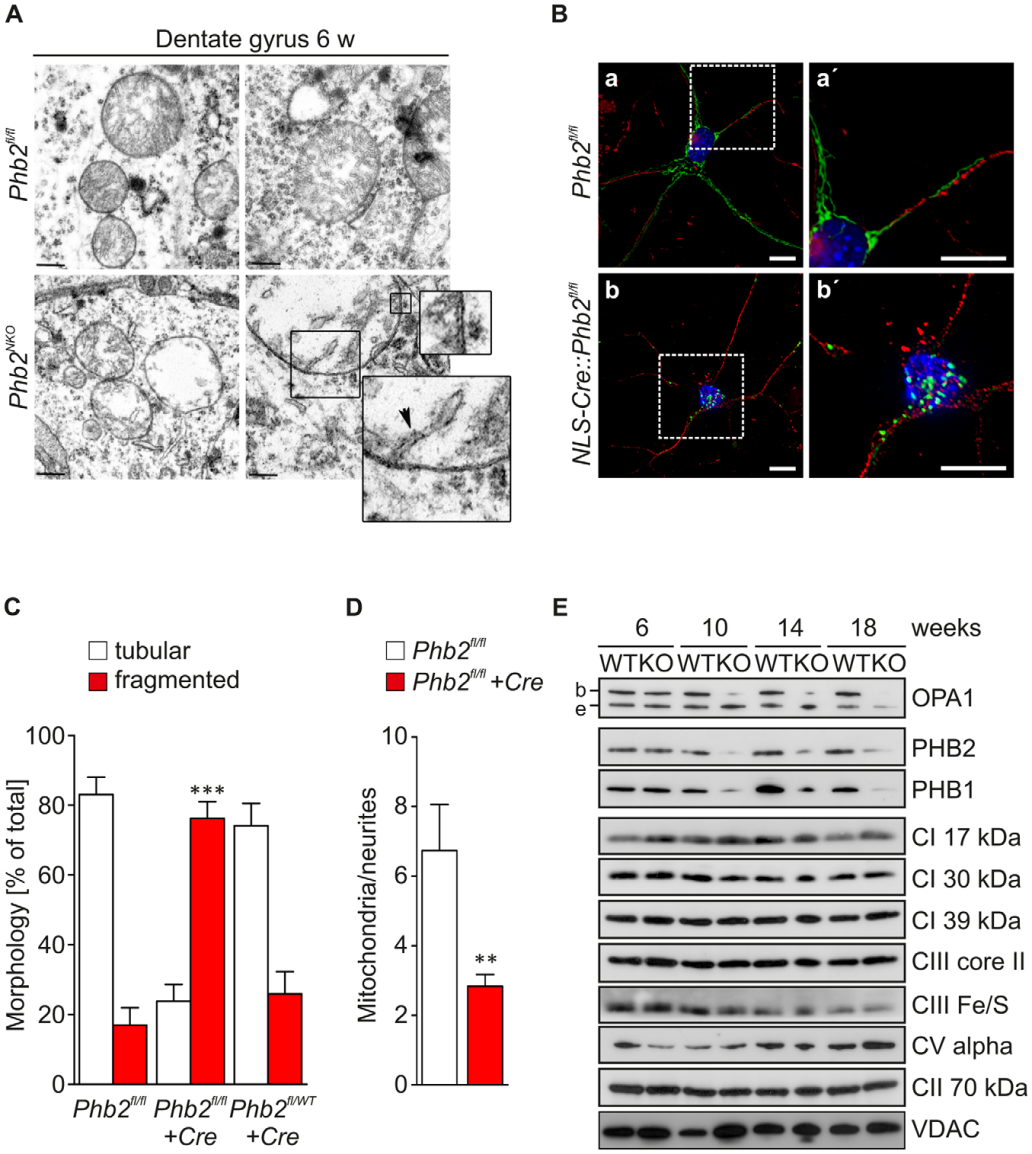
Defective mitochondrial morphogenesis and ultrastructure in *Phb2*-deficient neurons *in vivo*. (A) Transmission electron microscopy analysis of the mitochondrial ultrastructure in DG neurons of 6-week-old *Phb2^NKO^* and *Phb2^fl/fl^* control mice. The enlargements show the double membrane of the mitochondrion and the emergence of one crista. Scale bar: 400 nm. (B) Fragmentation and perinuclear clustering of PHB2-deficient neuronal mitochondria. Primary hippocampal neurons isolated from E18.5 *Phb2^fl/fl^* embryos were infected with lentiviruses expressing mitochondrially targeted EGFP and Cre recombinase (NLS-Cre) as indicated. Fixed samples were immunostained with antibodies directed against GFP and neuronal *β*III-tubulin followed by DAPI staining. a′, b′ are magnifications of the boxed insets shown in a, b. Scale bars: 10 µm. (C) Quantification of mitochondrial morphology in PHB2-deficient and control primary hippocampal neurons. Cells were infected with lentiviruses expressing Cre recombinase when indicated and processed as described in (B). Cells containing tubular (white bars) or fragmented mitochondria (red bars) were classified. >200 cells were scored in three independent experiments. ***P<0.001. Error bars indicate SEM. (D) Quantification of mitochondria per neurites in PHB2-deficient primary hippocampal neurons. *Phb2^fl/fl^* neurons were infected with lentiviruses expressing Cre recombinase when indicated and processed as described in (B). >30 cells were scored in three independent experiments. **P<0.01. Error bars indicate SEM. (E) Immunoblot analysis of hippocampal tissue lysates from *Phb2^NKO^* (KO) and *Phb2^fl/fl^* (WT) control mice of the indicated age. Lysates were analyzed by SDS-PAGE and immunoblotting using the indicated antibodies. Antibodies directed against VDAC and the 70 kDa subunit of complex II were used to monitor equal gel loading. b/e: long/short OPA1 isoforms.

To define whether the depletion of PHB2 affects mitochondrial ultrastructure in neurons at early stages of the pathological process, we analyzed the DG of young *Phb2^NKO^* mice by transmission electron microscopy. DG neurons of 6-week-old *Phb2^fl/fl^* control mice contained mitochondria with a normal appearance characterized by lamellar-shaped cristae inside double-membrane layered organelles (Figure 3A). In contrast, several neurons in the DG of *Phb2^NKO^* mice contained mitochondria with almost complete absence of lamellar cristae (Figure 3A). Moreover, in some cases these mitochondria appeared moderately swollen. These ultrastructural features account for the appearance of vacuolated neurons observed in semithin sections (Figure 2C). The number of neurons containing mitochondria with defective ultrastructure was further enhanced in 14-week-old animals confirming the progressive nature of this pathology (not shown).

To further investigate whether lack of PHB2 affects the mitochondrial network in neurons in a cell-autonomous manner, we isolated primary hippocampal neurons from conditional E18.5 *Phb2^fl/fl^* and *Phb2^fl/WT^* embryos and infected them with lentiviruses expressing nuclear-targeted Cre recombinase to genetically inactivate *Phb2 in vitro*. The mitochondrial network in these neurons was visualized by the simultaneous infection with lentiviral particles encoding a mitochondrially targeted EGFP (Su9-EGFP). Tubular mitochondria were present in the cell body and along the neurites in *Phb2^fl/fl^* (Figure 3B; a, a′) and *Phb2^fl/WT^* neurons, which were infected with Cre-expressing lentiviruses (*NLS-Cre::Phb2^fl/WT^*) (Figure 3C). In contrast, mitochondria were greatly fragmented and clustered in perinuclear regions of >70% of infected *Phb2^fl/fl^* neurons (*NLS-Cre::Phb2^fl/fl^*) (Figure 3B; b, b′). We further evaluated the mitochondrial distribution in *Phb2*-depleted neurons and determined the total number of mitochondria protruding into the neurites. Strikingly, neurites of Cre-infected *Phb2^fl/fl^* neurons contained fewer mitochondria when compared to controls consistent with the perinuclear clustering of fragmented mitochondria after acute loss of prohibitins (Figure 3D).

Different isoforms of the dynamin-like GTPase OPA1 with seemingly varying activities exist, which are expressed in a tissue-specific manner in mice [38]. The expression of OPA1 isoform 1 predominates in the central nervous system giving rise to bands b (L-OPA1) and, upon proteolytic processing, to band e (S-OPA1) [38]. To examine whether depletion of PHB2 affects the accumulation of OPA1 in neuronal tissue *in vivo*, we analyzed *Phb2^NKO^* and control forebrain lysates by immunoblotting with OPA1-specific antibodies. The loss of prohibitins was accompanied by the selective loss of the L-OPA1 isoform b in the hippocampus (Figure 3E), cortex and striatum but not in the cerebellum (Figure S7). These alterations occurred in a time-dependent manner simultaneous with the depletion of prohibitins and were already detected at 10 weeks of age. This does not reflect a general impairment of the biogenesis of mitochondrial inner membrane proteins, as various subunits of respiratory chain complexes accumulated at similar levels in the brain of *Phb2^NKO^* and control animals (Figure 3E; Figure S7). Overall, these data demonstrate that neuronal PHB2 ensures stabilization of L-OPA1 and the maintenance of the mitochondrial network and ultrastructure *in vivo*.

### Tau hyperphosphorylation in PHB2-deficient neurons

**Figure 4:**
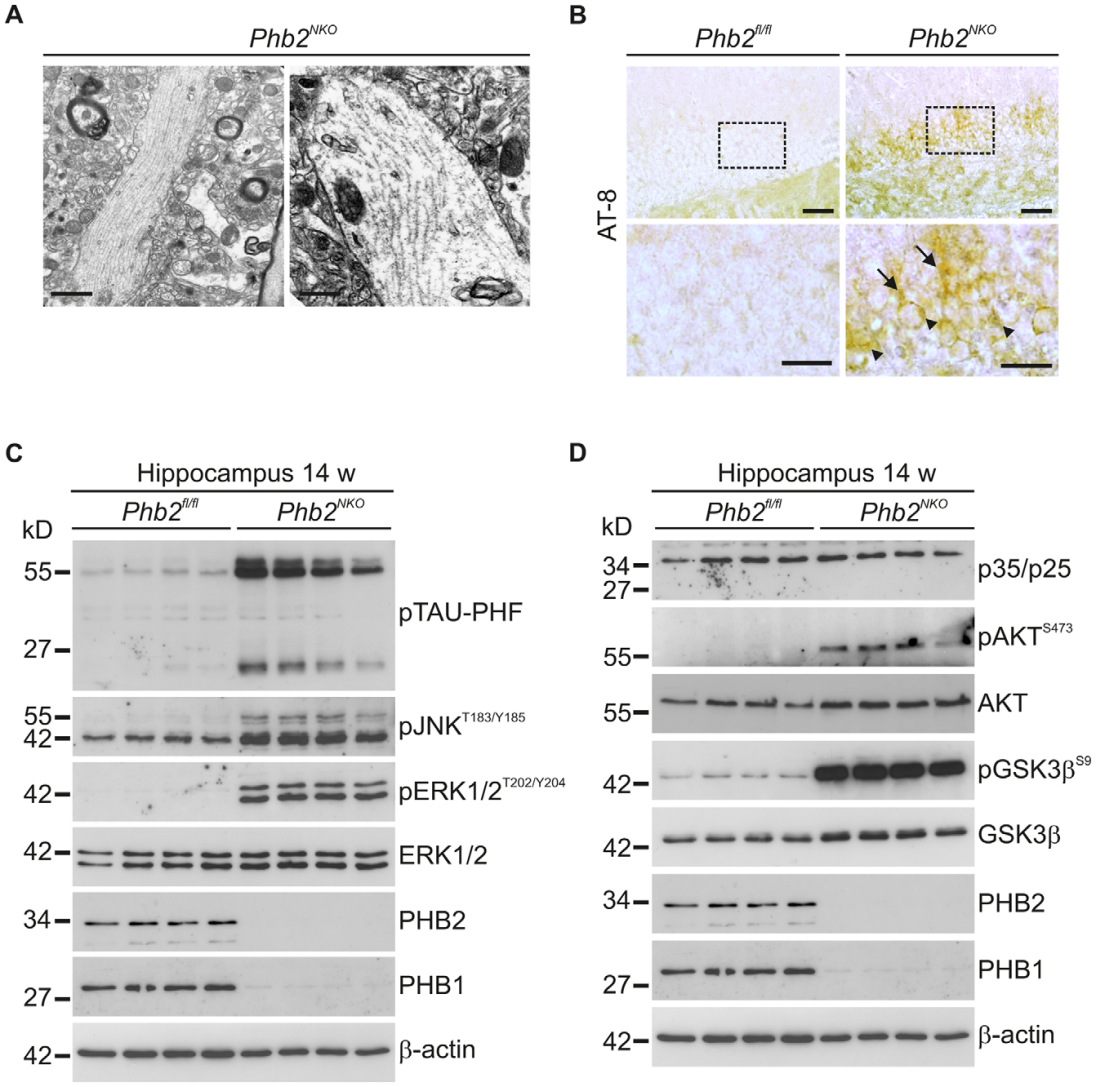
Tau hyperphosphorylation and filaments in *Phb2^NKO^* mice. (A) Transmission electron microscopy analysis of hippocampal tissue from 14-week-old *Phb2^NKO^* mice revealed the presence of straight filamentous tubules in neuronal unmyelinated processes reminiscent of tau filaments. Scale bars: 1.5 µm (left panel); 1 µm (right panel). (B) Immunohistochemistry using anti-AT8 antibody detecting hyperphosphorylated tau specifically on hippocampal tissue sections from 6-week-old *Phb2^NKO^* mice. Hyperphosphorylated tau accumulated both in the cell body (arrow head) and in dendrites (arrow) of DG neurons. The lower panel illustrates magnifications of the boxed insets depicted in the upper panel. Scale bars: 100 µm (upper panel), 50 µm (lower panel). (C) Immunoblot analysis of tau hyperphosphorylation and associated signalling molecules. Hippocampal tissue lysates from individual 14-week-old *Phb2^NKO^* and *Phb2^fl/fl^* control mice were analyzed by SDS-PAGE and immunoblotting using the indicated antibodies. β-actin was used as loading control. (D) Immunoblot analysis of signalling components that have been linked functionally to tau hyperphosphorylation. Hippocampal lysates were analyzed as in (C) using the indicated antibodies. β-actin was used as loading control.

Surprisingly, ultrastructural examination of hippocampi of 14-week-old *Phb2^NKO^* mice revealed the accumulation of straight tubular structures in unmyelinated neuronal processes. These filamentous structures measure about 12–20 nm in diameter (mean 20.8 nm±0.323; range 9.9–25.72 nm) and are reminiscent of inclusions composed of aberrantly phosphorylated species of the microtubule-associated protein tau. Although morphologically distinct from paired helical filaments (PHF), they are similar to those found in ‘classical’ intracytoplasmic inclusions of tau-positive astrocytes and neurons, which are observed in several neurodegenerative conditions such as frontotemporal dementia and other tauopathies (Figure 4A) [39].

To explore a role for *Phb2* in tau phosphorylation, hippocampal tissue sections were immunostained with AT-8 antibodies, which selectively recognize phosphorylated species of tau (phospho-Ser202 and phospho-Thr205). Intraneuronal inclusions were detected in the DG but not in other hippocampal regions of *Phb2^NKO^* mice as early as at 6 weeks but not in control littermates, and accumulated in both cell body and neurites (Figure 4B). We substantiated these observations by immunoblotting using phospho-tau specific AT-8 antiserum (Figure 4C). Several hyperphosphorylated tau species selectively accumulated in hippocampal lysates from 14-week-old *Phb2^NKO^* mice, but not in lysates from control mice (Figure 4C).

Several kinases have been implicated in tau phosphorylation both *in vitro* and *in vivo* [40], [41]. We therefore assessed the activation status of candidate kinases by immunoblotting of hippocampal extracts of *Phb2^NKO^* mice. Phosphorylated, active forms of the extracellular signal-regulated MAP kinases ERK1/2 and of the c-Jun N-terminal kinase JNK were detected specifically in *Phb2^NKO^* mice (Figure 4C). In contrast, the β-form of glycogen synthase kinase (GSK3), another putative major tau kinase, was robustly inactivated by phosphorylation at Ser position 9 (Figure 4D). Concomitantly, this was accompanied by the parallel activation of the upstream kinase AKT suggesting that the AKT-GSK3 axis might not be causative for the increased tau pathology in *Phb2^NKO^* mice (Figure 4D). Similarly, cyclin-dependent kinase 5 (CDK5) apparently does not contribute to tau hyperphosphorylation in *Phb2^NKO^* mice as we did not detect proteolytic conversion of its substrate p35 to p25 in *Phb2*-deficient hippocampal lysates (Figure 4D).

Taken together, we conclude from these experiments that deletion of *Phb2* activates MAP kinases leading to tau hyperphosphorylation and the deposition of aberrant filamentous structures in hippocampal neurons.

### Late-onset mitochondrial dysfunction and selective mtDNA loss in *Phb2^NKO^* tissues

**Figure 5:**
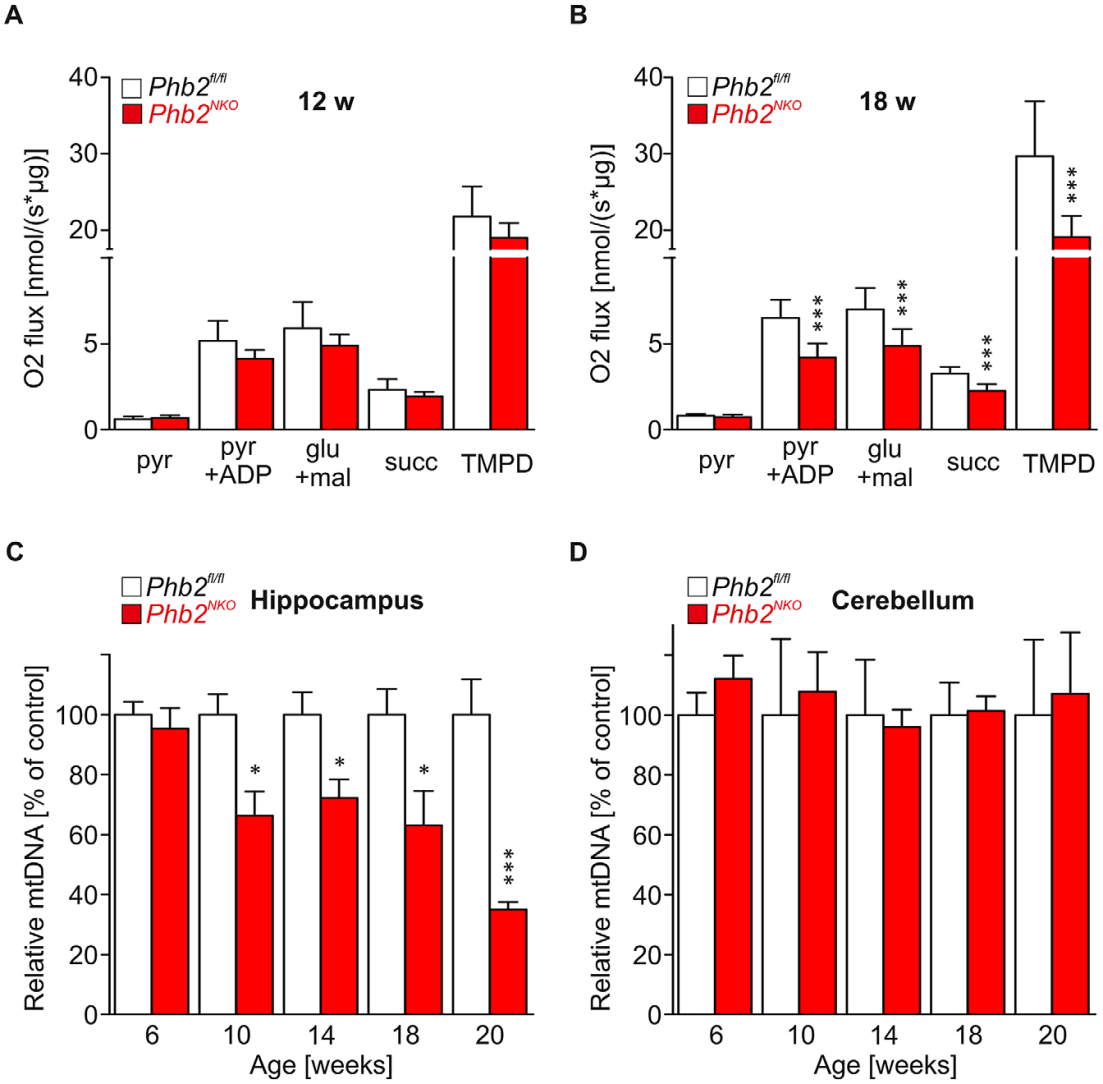
Late-onset mtDNA loss and respiratory dysfunction in *Phb2^NKO^* mice. (A) and (B) Oxygen consumption of mitochondria isolated from the hippocampus of (A) 12-week-old or (B) 18-week-old *Phb2^NKO^* and *Phb2^fl/fl^* control mice in the presence of specific substrates for individual respective respiratory chain complexes. Pyr, pyruvate; ADP, adenosine diphosphate; glu, glutamate; mal, malate; succ, succinate; TMPD, *N*,*N*,*N′*,*N′*-tetramethyl-1,4-phenylendiamine. n = 5. Error bars represent SEM. ***P<0.001. (C) and (D) Relative levels of mtDNA in the hippocampus (D) and the cerebellum (E) of *Phb2^NKO^* and *Phb2^fl/fl^* control mice. Total DNA was extracted from brain subregions of mice of the indicated age and genotype and analyzed by quantitative real-time PCR analysis using primers specific for mtDNA and nuclear DNA. Data represent average of at least three independent experiments, each sample assayed in quadruples. mtDNA, mitochondrial DNA. Error bars represent SEM. *P<0.05, ***P<0.001.

Mitochondrial dysfunction is an early phenomenon in many human tauopathies [42], [43]. To examine whether compromised mitochondrial respiratory function might be the underlying defect causing tau pathology and neurodegeneration in *Phb2^NKO^* mice, we monitored respiratory activities *in situ* and in isolated PHB2-deficient brain mitochondria. Enzymatic COX/SDH stainings on whole brain cryosections of 6-week-old *Phb2^NKO^* brains did not provide evidence for the presence of respiratory deficient cells (Figure S8). Consistently, substrate-driven respiration was not affected in mitochondria that had been isolated from hippocampal tissues of 12-week-old *Phb2^NKO^* mice (Figure 5A). Consistently, we obtained no evidence for increased ROS production and oxidative damage in 14-week-old *Phb2^NKO^* mice (Figure S9).

While not apparent in young mice, OXPHOS activities declined with age and were decreased significantly in 18-week-old *Phb2^NKO^* mice (Figure 5B). Mitochondria isolated from hippocampi of these mice were generally able to consume oxygen, as the basal mitochondrial respiration in the presence of pyruvate was similar in 18-week-old *Phb2^NKO^* and control mitochondria. However, respiration rates in PHB2-deficient mitochondria decreased significantly in the presence of saturating concentrations of ADP to maximally stimulate respiration, indicating that coupling is impaired in mitochondria depleted of PHB2. Moreover, enzymatic activities of complex I (monitored in the presence of glutamate and malate), complex II (in the presence of succinate) and of complex IV [in the presence of TMPD (*N*,*N*,*N′*,*N′*-Tetramethyl-1,4-phenylendiamine)] were significantly reduced in mitochondria isolated from 18-week-old *Phb2^NKO^* mice suggesting that respiratory activities in hippocampal tissues progressively deteriorate over time in the absence of PHB2 (Figure 5B).

The broad functional impairment of respiratory complexes in aged PHB2-deficient mice could be explained by a loss of the mitochondrial genome (mtDNA), which encodes essential respiratory chain subunits. We therefore determined mtDNA levels by quantitative real-time PCR analysis of mtDNA isolated from several neuronal tissues of *Phb2^NKO^* and control mice. Strikingly, mtDNA levels relative to nuclear DNA deteriorated in a progressive manner in the hippocampus and striatum but not in the cerebellum of *Phb2^NKO^* mice (Figure 5C, 5D, Figure S10). In 20-week-old *Phb2^NKO^* animals, relative mtDNA levels were reduced to 30% of controls in the hippocampus, providing a rationale for the decreased respiratory activities in these mice. It is noteworthy that mtDNA levels were not affected in cortical PHB2-deficient mitochondria (Figure S10), pointing to neuronal-specific differences in the mechanisms that stabilize mtDNA.

In conclusion, these experiments demonstrate that PHB2 is required for the maintenance of mtDNA in neuronal mitochondria. The loss of PHB2 in the forebrain leads to a progressive destabilization of mtDNA and ultimately to an impaired respiratory function. However, respiratory deficiencies become apparent at significantly later stages than tau phosphorylation suggesting that they are not the primary cause for the tau pathology in PHB2-deficient mice.

## Discussion

Our analysis of *Phb2^NKO^* mice unravelled essential functions of prohibitins for the survival of adult neurons *in vivo*. Impaired OPA1 processing and hyperphosphorylation of tau manifest early during this degeneration process. Our observations therefore establish the requirement of prohibitins for mitochondrial fusion and ultrastructure in neurons and provide a novel model for tau pathologies induced by mitochondrial dysfunctions.

### Prohibitins are required for neuronal survival

We observe massive degeneration of PHB2-deficient neurons in the forebrain. Neurons expressing Cre recombinase are lost or severely affected in *Phb2^NKO^* mice, demonstrating a general requirement of prohibitins for neuronal survival *in vivo*. TUNEL stainings of DG neurons suggest that apoptosis contributes to neuronal loss but other types of cell death cannot be excluded. Consistently, depletion of prohibitins was found to facilitate apoptosis in different cell types *in vitro* [13], [44], [45]. It is noteworthy that the susceptibility towards apoptosis appears to vary between different cell types [13], [44], [45]. Similarly, the loss of PHB2 in *Phb2^NKO^* mice leads to faster death of DG neurons when compared to CA1 neurons, pointing to neuron-specific differences.

The loss of hippocampal neurons in *Phb2^NKO^* mice is associated with anxiolytic behavior and deficiencies in memory function and in learning abilities. Moreover, *Phb2^NKO^* mice develop progressive cachexia and kyphosis. In view of massive neuronal loss in the hippocampal region of *Phb2^NKO^* mice, it appears likely that reduced food intake causes these phenotypes. As *Phb2* might only be partially deleted in the hypothalamic region of *Phb2^NKO^* mice [36], it remains to be determined how the loss of PHB2 in the forebrain causes these phenotypes, which are reminiscent of other mouse lines harboring dysfunctional mitochondria [46], [47]. Regardless, they are likely the consequence of the massive neuronal loss in *Phb2^NKO^* mice rather than reflecting specific functions of prohibitins in the forebrain.

### Prohibitins as mitochondrial membrane scaffolds in neurons

Ring complexes formed of multiple PHB1 and PHB2 subunits act as scaffolds in the inner membrane affecting the spatial organization of membrane proteins and lipids [16], [48]. Previous studies in proliferating cells *in vitro* revealed that prohibitin complexes ensure the accumulation of L-OPA1 within mitochondria [13]. We now extend these findings to adult neurons *in vivo* and establish an essential role of prohibitins for the maintenance of mitochondrial ultrastructure. Destabilization of L-OPA1 in the absence of PHB2 likely inhibits fusion and ongoing fission events lead to the fragmentation of the mitochondrial network in hippocampal neurons. Moreover, we demonstrate that prohibitin scaffolds are required to maintain the mitochondrial genome, which is progressively lost in neurons lacking PHB2 and likely explains respiratory deficiencies that occur in aged PHB2-deficient neurons. Notably, mtDNA is absent in fusion-incompetent mitochondria in MFN2-deficient fibroblasts [5], indicating that mitochondrial fusion or the protein machinery involved is required to maintain mtDNA. It is therefore conceivable that neurons lacking PHB2 lose mtDNA because mitochondrial fusion is inhibited. Alternatively, PHB2 acting as a membrane scaffold may directly affect the stability of mitochondrial nucleoids in neurons. Prohibitins have been identified as peripheral components of mitochondrial nucleoids and were found to maintain their organization and stability at least in some cell lines *in vitro* [14], [49]. In yeast, depletion of prohibitins in combination with components affecting the accumulation of phosphatidyl ethanolamine in mitochondrial membranes induces the loss of the mitochondrial genome [19], [50], supporting a critical role of the membrane environment for the maintenance of mtDNA.

Taken together, our observations demonstrate that neuronal survival *in vivo* critically depends on prohibitin scaffolds in the inner membrane and identify the processing of OPA1 and the stability of the mitochondrial genome as processes within mitochondria, whose perturbation leads to neurodegeneration in the absence of prohibitins.

### Loss of PHB2 causes tau hyperphosphorylation and neurodegeneration

Our findings also provide insight into the cellular mechanisms through which a dysfunction of mitochondria leads to neurodegeneration. The observation of impaired OPA1 processing and defective mitochondrial ultrastructure preceding massive neuronal loss in *Phb2^NKO^* mice supports emerging evidence that neurons are particularly susceptible to perturbations in mitochondrial dynamics. Studies on the cerebellum of MFN2-deficient mice revealed electron transport deficiencies of Purkinje cells prior to neuronal death, which are consistent with the lack of mtDNA nucleoids observed in fibroblasts [5]. The dependence of mtDNA stability and respiratory activity on mitochondrial fusion provides an elegant mechanism to explain neuronal loss in MFN2-deficient mice [5]. However, while the lack of PHB2 destabilizes mtDNA in the hippocampus and striatum, respiratory deficiencies manifest only in aged *Phb2^NKO^* mice, indicating that alternative mechanisms lead to neurodegeneration in this model.

The analysis of mitochondrial morphology in PHB2-deficient hippocampal neurons suggests that deficiencies in mitochondrial distribution may trigger neuronal loss. Fragmented mitochondria accumulate in the perinuclear region of hippocampal neurons lacking PHB2 *in vitro* and are depleted from neurites. The surprising observation of tau hyperphosphorylation and aggregation provides a possible explanation for the altered distribution of mitochondria in PHB2-deficient neurons. Consistent with an important role for neurodegeneration, we detected tau phosphorylated at AT-8 sites already in 6-week-old *Phb2^NKO^* mice, i.e. before neuronal loss becomes apparent. Tau is predominantly present in axons, where it binds and stabilizes microtubules and regulates axonal transport processes [43], [51], [52]. Hyperphosphorylated forms of tau were found to detach from microtubules, accumulate in the soma and are prone to aggregation. Consistently, phosphorylated tau was found to interfere with the binding of kinesin motors to mitochondria and distinct vesicles affecting cargo-selective anterograde transport in cultured neurons [52]. Moreover, phosphorylation of tau at AT-8 sites was recently found to modulate mitochondrial movement in cortical neurons [53]. It is therefore conceivable that tau hyperphosphorylation in the absence of PHB2 causes mitochondrial transport deficiencies triggering progressive neuronal loss in *Phb2^NKO^* mice.

Hyperphosphorylation of tau has been observed in AD brains [54]. Stress-activated kinases like JNK and ERK1/2 have been implicated in the hyperphosphorylation of tau during AD. In fact, fibrillar Aβ can induce ERK activation, abnormal phosphorylation of Tau, and progressive neurodegeneration [55]. In addition, JNK-related kinases are activated in AD brains and are associated with the development of amyloid plaques [56]. However, despite extensive studies on tau hyperphosphorylation, the complexity of kinases and phosphatases involved has precluded to define its pathogenic role for AD until now [57].

Regardless, the discovery of tau hyperphosphorylation and filament formation upon loss of PHB2 sheds new light on the possible role of mitochondria in neurodegeneration in AD and related disorders. While mitochondrial dysfunction has been recognized as a prominent, early event in a number of tauopathies including AD [51], it remained open whether mitochondrial defects are of direct pathogenic relevance or secondary to other cellular deficiencies. Our analysis of *Phb2^NKO^* mice provides first genetic evidence that a dysfunction of mitochondria can trigger tau hyperphosphorylation and aggregation. We detected phosphorylated tau in PHB2-deficient hippocampal neurons lacking apparent respiratory defects or evidence for oxidative damage strongly suggesting that other mechanisms induce tau pathologies in this model. Perturbations in mitochondrial dynamics and ultrastructure that occur early in *Phb2^NKO^* mice and may interfere with axonal trafficking are attractive candidates. Our findings therefore raise the possibility that tau pathologies might be associated with other neurodegenerative disorders caused by deficiencies in mitochondrial dynamics. Studies along these lines may turn out to be of relevance for tauopathies as well.

## Materials and Methods

### Histology and immunohistochemistry

Animals were anesthetized with avertin and perfused intracardially with 4% paraformaldehyde in PBS. Brain were removed, post-fixed overnight with 4% paraformaldehyde in PBS and conserved in 0.12 M phosphate buffer. Immunohistochemistry and immunofluorescence were performed on 30 µm sagittal vibratome sections, as previously described [58]. Anti-GFAP antibodies were purchased by NeoMarkers (Fremont, CA, USA). Anti-4-HNE antibodies were purchased from Abcam (Cambridge, UK). Immunohistochemistry with anti-AT-8 (Thermo Fisher Scientific,Walthman, MA, USA) was performed with Vector M.O.M. Immunodetection kit (Vector Lab, Burlingame, CA, USA) according to the manufacturer's protocol. For TUNEL assays, tissues were frozen on liquid nitrogen vapour for 5 s after fixation and then conserved in liquid nitrogen. TUNEL assays were performed on 20 µm thick coronal frozen sections with ApopTag Plus Peroxidase *In Situ* Apoptosis Detection Kit (Chemicon International Temecula, CA) according to the manufacturer's protocol. All immunohistochemical and immunofluorescence analyses were performed on at least three mice per genotype.

### Neuropathology and ultrastructural analysis

Age-matched *Phb2^NKO^* and control mice (n = 3 for each genotype) were anesthetized intraperitoneally with avertin and perfused with 2% glutaraldehyde in PBS. Brains were removed and postfixed in 0.12 M phosphate buffer/2% glutaraldehyde. After treatment with osmium tetroxide, brains were embedded in Epon (Fluka, Buchs SG, Switzerland). Semithin (1 µm) coronal sections were cut from hippocampus and cerebral cortex. To quantify the number of DG neurons with degenerative features, we performed morphometry on semithin sections by scoring the percentage of DG neurons with abnormal morphology and vacuoles in the cytoplasm, and by counting the number of neurons in the DG and CA1 areas (n = 3 per genotype). Morphometric analyses were performed blinded to the mouse genotype. For ultrastructural analyses, blocks of tissue were selected for electron microscopy after light microscopy examination of semithin sections. Ultrathin sections (70 nm) were cut, collected on 200 mesh copper grids (Electron Microscopy Sciences, Hatfield, PA, USA) and stained with uranium acetate (Plano GMBH, Wetzlar, Germany) and lead citrate (Electron Microscopy Sciences).

### RNA in situ hybridization

To obtain specific probes for in situ hybridization, the coding sequence of the mouse *Phb2* (nucleotides 1–900) cDNA was PCR-amplified from mouse liver cDNA, subcloned and used as templates to transcribe either sense or antisense digoxygenin-labeled riboprobes using the DIG RNA labeling kit (Roche). Vibratome sections were permeabilized with proteinase K (10 µg/ml) for 10 min. In situ hybridization was performed essentially as described previously [59].

### Enzyme activity staining of brain cryosections

Frozen brain cryosections were thawed and incubated in COX staining solution (DAB, cytochrome *c*, sucrose, catalase, phosphate buffer pH 7.4), SDH staining solution (succinic acid, phosphate buffer pH 7.4) or both in a humid chamber for 15 min at 37°C. Slides were washed three times with water for 5 min. For dehydration samples were incubated in increasing concentrations of ethanol: 90% EtOH for 1 min, 95% EtOH for 1 min and 100% EtOH for 1 min. Subsequently, the sections were washed two times in xylol for 2 min each and finally mounted in mounting medium.

### Primary neuronal cultures

Mouse primary hippocampal neurons were isolated from E18.5 embryos (*Phb2^fl/fl^* and *Phb2^fl/wt^*) and grown on coverslips for 7 DIV before transduction with lentiviral vectors. Detailed experimental procedures are found in the supplement.

## Supporting Information

Figure S1 Spatially restricted Cre-recombination in mice expressing Cre recombinase under the control of the *CaMKIIα* promoter. β-galactosidase activity staining of parasagittal (a, d) and coronal sections (b, c) of *CaMKIIa-Cre/ROSA26-lacZ* reporter brains revealed spatially-restricted Cre recombination in the cortex (CO), the striatum (ST), the hippocampus (HC) and the hypothalamus (d). Maximal recombination efficiency was observed in the hippocampus, in which all neuronal compartments [cornu ammonis (CA), dentate gyrus (DG)] showed strong β-galactosidase staining. CB = cerebellum. Scale bars: 1 mm (a, b); 0,5 mm (c, d).
(PDF)

Figure S2 Whole-body CT scans of *Phb2^NKO^* mice. (A) and (B) Representative Micro-CT scans of 21-week-old (A) male and (B) female *Phb2^NKO^* and *Phb2^HET^* control mice. *Phb2^NKO^* mice displayed a strong curvature of the spinal column (lordokyphosis) and reduction of body size and mass.
(PDF)

Figure S3 Impaired learning and memory abilities of *Phb2^NKO^* mice. (A) Escape latencies of 8-week-old *Phb2^NKO^* (n = 12) and *Phb2^fl/fl^* control mice (n = 13) were examined with the Morris water maze hidden platform paradigm during a 5-day training period. ***P<0.001. Error bars indicate SEM. (B) Swim path comparisons of 8-week-old *Phb2^NKO^* (n = 12) and *Phb2^fl/fl^* (n = 13) control mice assessed during the training phase in the Morris water maze on five consecutive days. The total distance travelled in four trials per training day is indicated. *P<0.05; **P<0.01; ***P<0.001. Error bars indicate SEM. (C) Swimming times of 8-week-old *Phb2^NKO^* (n = 12) and *Phb2^fl/fl^* control mice (n = 13) spent in each quadrant in the probe trial on day 5. The dotted line indicates the chance level (25%). ***P<0.001. Error bars indicate SEM. (D) Representative path tracings of 8-week-old *Phb2^NKO^* and *Phb2^fl/fl^* control mice during the probe trial on day 5. The coloured quadrant indicates the target region after removal of the platform. (E) Swim path comparisons of *Phb2^NKO^* mice and *Phb2^fl/fl^* controls assessed during the probe trial in the Morris water maze on day 5. Values are expressed as the total distance travelled during 60 s of the probe trial. ***P<0.001. Error bars indicate SEM. (F) Swim velocities of 8-week-old *Phb2^NKO^* (n = 12) and *Phb2^fl/fl^* (n = 13) control mice assessed during the probe trial in the Morris water maze on day 5. The total distance travelled per 60 sec during the probe trial is indicated. Error bars indicate SEM.
(PDF)

Figure S4 Reduced anxiety and loss of motor coordination in *Phb2^NKO^* mice. (A) Elevated zero maze analysis of 8-week-old *Phb2^NKO^* (n = 12) and *Phb2^fl/fl^* control mice (n = 13). Values are expressed as percentage of time spent in either open or closed areas of the maze. **P<0.01. Error bars indicate SEM. (B) Total distance of *Phb2^NKO^* (n = 12) and *Phb2^fl/fl^* control mice (n = 13) travelled in the elevated zero maze (EZM). **P<0.01. Error bars indicate SEM. (C) Open field test of 8-week-old *Phb2^NKO^* (n = 12) and *Phb2^fl/fl^* control mice (n = 13). Values are expressed as percentage of time spent in the center of the open field. ***P<0.001. Error bars indicate SEM. (D) Vertical locomotion of 8-week-old *Phb2^NKO^* (n = 12) and *Phb2^fl/fl^* (n = 13) control mice assessed from total rearing events during a 5-minute test phase in the open field paradigm. ***P<0.001. Error bars indicate SEM. (E) Total distance of *Phb2^NKO^* (n = 12) and *Phb2^fl/fl^* control mice (n = 13) travelled in the open field. ***P<0.001. Error bars indicate SEM. (F) Locomotor activity of 8-week-old *Phb2^NKO^* and *Phb2^fl/fl^* control mice during day-night cycle measured in metabolic cages. Data represent total beam break counts during a 12 hour period. n = 4 per group. ***P<0.001. Error bars indicate SEM. (G) Representative photographs of pathological hindlimb clasping reflexes during tail suspension in 18-week-old *Phb2^NKO^* mice (lower panel) compared to *Phb2^fl/fl^* controls (upper panel). (H) Rotarod performance test of *Phb2^NKO^* (n = 12) and *Phb2^fl/fl^* control mice (n = 13) examined at the indicated time points. *P<0.05; ***P<0.001. Error bars indicate SEM.
(PDF)

Figure S5 Detection of apoptotic DG neurons in *Phb2^NKO^* mice. TUNEL staining of DG neurons in 6-week-old *Phb2^NKO^* mice is shown (black arrows). Scale bar: 20 µm.
(PDF)

Figure S6 Extensive loss of hippocampal and cortical neurons in *Phb2^NKO^* mice. (A) Loss of pyramidal neurons in all hippocampal layers of 20-week-old *Phb2^NKO^* mice. Coronal semithin sections of the indicated *cornu ammonis* (CA) areas (CA1, CA2 and CA3) from 20-week-old *Phb2^NKO^* and *Phb2^fl/fl^* control mice. Scale bars: 20 µm. (B) Late-onset morphological alterations of cerebral cortex neurons in 20-week-old *Phb2^NKO^* mice. Coronal semithin sections of cerebral cortex from layers I to VI of 20-week-old *Phb2^NKO^* and *Phb2^fl/fl^* control mice. Scale bars: 20 µm.
(PDF)

Figure S7 Immunoblot analysis of forebrain tissue lysates of *Phb2^NKO^* mice. Tissue lysates from cortex, striatum und cerebellum of *Phb2^NKO^* (KO) and *Phb2 ^fl/fl^* (WT) control mice of the indicated age were analyzed by SDS-PAGE and immunoblotting using the indicated antibodies. Antibodies directed against VDAC and the 70 kDa subunit of complex II were used to monitor equal gel loading. b/e: long/short OPA1 isoforms.
(PDF)

Figure S8 COX and SDH activities in DG neurons of 6-week-old *Phb2^NKO^* mice. Cross-sections of coronal brain regions from 6-week-old *Phb2^NKO^* and *Phb2^fl/fl^* control mice were stained for either COX or SDH activities or for both. Representative micrographs are shown. Scale bar: 40 µm.
(PDF)

Figure S9 Monitoring oxidative damage in *Phb2^NKO^* mice. Hippocampal lysates of 14-week-old *Phb2^NKO^* and *Phb2^fl/fl^* control mice were analyzed by SDS-PAGE and immunoblotting using the indicated antibodies. β-actin was used as a loading control. 4-hydroxynonenal (4-HNE) stainings of coronal sections of the DG of 14-week-old *Phb2^NKO^* and *Phb2^fl/fl^* control mice did not reveal any signs of lipid oxidation (data not shown).
(PDF)

Figure S10 Tissue-specific mtDNA loss in PHB2-deficient neurons *in vivo*. (A) and (B) Relative levels of mtDNA in (A) striatum and (B) cortex of *Phb2^NKO^* and *Phb2^fl/fl^* control mice. Total DNA was extracted from brain subregions of mice of the indicated age and genotype and analyzed by quantitative real-time PCR analysis using primers specific for mtDNA and nuclear DNA. Data represent average of at least three independent experiments, each sample assayed in quadruples. mtDNA, mitochondrial DNA. Error bars represent SEM. **P<0.01.
(PDF)

Text S1 Supporting behavioral studies and supporting methods.
(DOCX)

## References

[1] Westermann B (2010) Mitochondrial fusion and fission in cell life and death. Nat Rev Mol Cell Biol 11: 872–884. 21102612 10.1038/nrm3013

[2] Chen H, Chan DC (2010) Physiological functions of mitochondrial fusion. Ann N Y Acad Sci 1201: 21–25. 20649534 10.1111/j.1749-6632.2010.05615.x

[3] de Brito OM, Scorrano L (2010) An intimate liaison: spatial organization of the endoplasmic reticulum-mitochondria relationship. The EMBO journal 29: 2715–2723. 20717141 10.1038/emboj.2010.177 PMC2924651

[4] Rugarli EI, Langer T (2012) Mitochondrial quality control: A matter of life and death for neurons. EMBO J in press. 10.1038/emboj.2012.38 PMC3321185 22354038

[5] Chen H, McCaffery JM, Chan DC (2007) Mitochondrial fusion protects against neurodegeneration in the cerebellum. Cell 130: 548–562. 17693261 10.1016/j.cell.2007.06.026

[6] Ishihara N, Nomura M, Jofuku A, Kato H, Suzuki SO, et al (2009) Mitochondrial fission factor Drp1 is essential for embryonic development and synapse formation in mice. Nature cell biology 11: 958–966. 19578372 10.1038/ncb1907

[7] Alexander C, Votruba M, Pesch UE, Thiselton DL, Mayer S, et al (2000) *OPA1*, encoding a dynamin-related GTPase, is mutated in autosomal dominant optic atrophy linked to chromosome 3q28. Nat Genet 26: 211–215. 11017080 10.1038/79944

[8] Delettre C, Lenaers G, Griffoin JM, Gigarel N, Lorenzo C, et al (2000) Nuclear gene *OPA1*, encoding a mitochondrial dynamin-related protein, is mutated in dominant optic atrophy. Nat Genet 26: 207–210. 11017079 10.1038/79936

[9] Züchner S, Mersiyanova IV, Muglia M, Bissar-Tadmouri N, Rochelle J, et al (2004) Mutations in the mitochondrial GTPase mitofusin 2 cause Charcot-Marie-Tooth neuropathy type 2A. Nat Genet 36: 449–451. 15064763 10.1038/ng1341

[10] Song W, Chen J, Petrilli A, Liot G, Klinglmayr E, et al (2011) Mutant huntingtin binds the mitochondrial fission GTPase dynamin-related protein-1 and increases its enzymatic activity. Nature medicine 17: 377–382. 10.1038/nm.2313 PMC3051025 21336284

[11] Su B, Wang X, Zheng L, Perry G, Smith MA, et al (2010) Abnormal mitochondrial dynamics and neurodegenerative diseases. Biochimica et biophysica acta 1802: 135–142. 19799998 10.1016/j.bbadis.2009.09.013 PMC2790543

[12] Knott AB, Perkins G, Schwarzenbacher R, Bossy-Wetzel E (2008) Mitochondrial fragmentation in neurodegeneration. Nat Rev Neurosci 9: 505–518. 18568013 10.1038/nrn2417 PMC2711514

[13] Merkwirth C, Dargazanli S, Tatsuta T, Geimer S, Lower B, et al (2008) Prohibitins control cell proliferation and apoptosis by regulating OPA1-dependent cristae morphogenesis in mitochondria. Genes Dev 22: 476–488. 18281461 10.1101/gad.460708 PMC2238669

[14] Kasashima K, Sumitani M, Satoh M, Endo H (2008) Human prohibitin 1 maintains the organization and stability of the mitochondrial nucleoids. Exp Cell Res 314: 988–996. 18258228 10.1016/j.yexcr.2008.01.005

[15] Sato S, Murata A, Orihara T, Shirakawa T, Suenaga K, et al (2011) Marine Natural Product Aurilide Activates the OPA1-Mediated Apoptosis by Binding to Prohibitin. Chem Biol 18: 131–139. 21276946 10.1016/j.chembiol.2010.10.017

[16] Osman C, Merkwirth C, Langer T (2009) Prohibitins and the functional compartmentalization of mitochondrial membranes. J Cell Sci 122: 3823–3830. 19889967 10.1242/jcs.037655

[17] Artal-Sanz M, Tavernarakis N (2010) Opposing function of mitochondrial prohibitin in aging. Aging 2: 1004–1011. 21164222 10.18632/aging.100246 PMC3034168

[18] Tatsuta T, Model K, Langer T (2005) Formation of membrane-bound ring complexes by prohibitins in mitochondria. Mol Biol Cell 16: 248–259. 15525670 10.1091/mbc.E04-09-0807 PMC539169

[19] Osman C, Haag M, Potting C, Rodenfels J, Dip PV, et al (2009) The genetic interactome of prohibitins: coordinated control of cardiolipin and phosphatidylethanolamine by conserved regulators in mitochondria. J Cell Biol 184: 583–596. 19221197 10.1083/jcb.200810189 PMC2654118

[20] Tavernarakis N, Driscoll M, Kyrpides NC (1999) The SPFH domain: implicated in regulating targeted protein turnover in stomatins and other membrane-associated proteins. Trends Biochem Sci 24: 425–427. 10542406 10.1016/s0968-0004(99)01467-x

[21] Browman DT, Hoegg MB, Robbins SM (2007) The SPFH domain-containing proteins: more than lipid raft markers. Trends Cell Biol 17: 394–402. 17766116 10.1016/j.tcb.2007.06.005

[22] Artal-Sanz M, Tsang WY, Willems EM, Grivell LA, Lemire BD, et al (2003) The mitochondrial prohibitin complex is essential for embryonic viability and germline function in *Caenorhabditis elegans* . J Biol Chem 278: 32091–32099. 12794069 10.1074/jbc.M304877200

[23] Park SE, Xu J, Frolova A, Liao L, O'Malley BW, et al (2005) Genetic deletion of the repressor of estrogen receptor activity (REA) enhances the response to estrogen in target tissues in vivo. Mol Cell Biol 25: 1989–1999. 15713652 10.1128/MCB.25.5.1989-1999.2005 PMC549370

[24] Artal-Sanz M, Tavernarakis N (2009) Prohibitin couples diapause signalling to mitochondrial metabolism during ageing in C. elegans. Nature 461: 793–797. 19812672 10.1038/nature08466

[25] Schleicher M, Shepherd BR, Suarez Y, Fernandez-Hernando C, Yu J, et al (2008) Prohibitin-1 maintains the angiogenic capacity of endothelial cells by regulating mitochondrial function and senescence. J Cell Biol 180: 101–112. 18195103 10.1083/jcb.200706072 PMC2213620

[26] Song Z, Chen H, Fiket M, Alexander C, Chan DC (2007) OPA1 processing controls mitochondrial fusion and is regulated by mRNA splicing, membrane potential, and Yme1L. J Cell Biol 178: 749–755. 17709429 10.1083/jcb.200704110 PMC2064540

[27] Ishihara N, Fujita Y, Oka T, Mihara K (2006) Regulation of mitochondrial morphology through proteolytic cleavage of OPA1. EMBO J 25: 2966–2977. 16778770 10.1038/sj.emboj.7601184 PMC1500981

[28] Griparic L, Kanazawa T, van der Bliek AM (2007) Regulation of the mitochondrial dynamin-like protein Opa1 by proteolytic cleavage. J Cell Biol 178: 757–764. 17709430 10.1083/jcb.200704112 PMC2064541

[29] Duvezin-Caubet S, Jagasia R, Wagener J, Hofmann S, Trifunovic A, et al (2006) Proteolytic processing of OPA1 links mitochondrial dysfunction to alterations in mitochondrial morphology. J Biol Chem 281: 37972–37979. 17003040 10.1074/jbc.M606059200

[30] Ehses S, Raschke I, Mancuso G, Bernacchia A, Geimer S, et al (2009) Regulation of OPA1 processing and mitochondrial fusion by m-AAA protease isoenzymes and OMA1. J Cell Biol 187: 1023–1036. 20038678 10.1083/jcb.200906084 PMC2806285

[31] Steglich G, Neupert W, Langer T (1999) Prohibitins regulate membrane protein degradation by the *m*-AAA protease in mitochondria. Mol Cell Biol 19: 3435–3442. 10207067 10.1128/mcb.19.5.3435 PMC84136

[32] Piechota J, Kolodziejczak M, Juszczak I, Sakamoto W, Janska H (2010) Identification and characterization of high molecular weight complexes formed by matrix AAA proteases and prohibitins in mitochondria of Arabidopsis thaliana. The Journal of biological chemistry 285: 12512–12521. 20172857 10.1074/jbc.M109.063644 PMC2857092

[33] Casari G, De-Fusco M, Ciarmatori S, Zeviani M, Mora M, et al (1998) Spastic paraplegia and OXPHOS impairment caused by mutations in paraplegin, a nuclear-encoded mitochondrial metalloprotease. Cell 93: 973–983. 9635427 10.1016/s0092-8674(00)81203-9

[34] DiBella D, Lazzaro F, Brusco A, Battaglia G, A P, et al (2008) AFG3L2 mutations cause autosomal dominant ataxia SCA28 and reveal an essential role of the m-AAA AFG3L2 homocomplex in the cerebellum. Annual meeting of the American Society of Human Genetics Philadelphia, Pennsylvania.

[35] Pierson TM, Adams D, Bonn F, Martinelli P, Cherukuri PF, et al (2011) Whole-exome sequencing identifies homozygous AFG3L2 mutations in a spastic ataxia-neuropathy syndrome linked to mitochondrial m-AAA proteases. PLoS Genet 7: e1002325 doi:10.1371/journal.pgen.1002325 22022284 10.1371/journal.pgen.1002325 PMC3192828

[36] Minichiello L, Korte M, Wolfer D, Kuhn R, Unsicker K, et al (1999) Essential role for TrkB receptors in hippocampus-mediated learning. Neuron 24: 401–414. 10571233 10.1016/s0896-6273(00)80853-3

[37] Soriano P (1999) Generalized lacZ expression with the ROSA26 Cre reporter strain. Nat Genet 21: 70–71. 9916792 10.1038/5007

[38] Akepati VR, Muller EC, Otto A, Strauss HM, Portwich M, et al (2008) Characterization of OPA1 isoforms isolated from mouse tissues. Journal of neurochemistry 106: 372–383. 18419770 10.1111/j.1471-4159.2008.05401.x

[39] Giaccone G, Marcon G, Mangieri M, Morbin M, Rossi G, et al (2008) Atypical tauopathy with massive involvement of the white matter. Neuropathology and applied neurobiology 34: 468–472. 18208485 10.1111/j.1365-2990.2007.00927.x

[40] Hanger DP, Anderton BH, Noble W (2009) Tau phosphorylation: the therapeutic challenge for neurodegenerative disease. Trends in molecular medicine 15: 112–119. 19246243 10.1016/j.molmed.2009.01.003

[41] Mazanetz MP, Fischer PM (2007) Untangling tau hyperphosphorylation in drug design for neurodegenerative diseases. Nature reviews Drug discovery 6: 464–479. 17541419 10.1038/nrd2111

[42] Schon EA, Przedborski S (2011) Mitochondria: the next (neurode)generation. Neuron 70: 1033–1053. 21689593 10.1016/j.neuron.2011.06.003 PMC3407575

[43] Reddy PH (2011) Abnormal tau, mitochondrial dysfunction, impaired axonal transport of mitochondria, and synaptic deprivation in Alzheimer's disease. Brain research 1415: 136–148. 21872849 10.1016/j.brainres.2011.07.052 PMC3176990

[44] Kasashima K, Ohta E, Kagawa Y, Endo H (2006) Mitochondrial functions and estrogen receptor-dependent nuclear translocation of pleiotropic human prohibitin 2. J Biol Chem 281: 36401–36410. 17008324 10.1074/jbc.M605260200

[45] Zhou P, Qian L, D'Aurelio M, Cho S, Wang G, et al (2012) Prohibitin reduces mitochondrial free radical production and protects brain cells from different injury modalities. The Journal of neuroscience: the official journal of the Society for Neuroscience 32: 583–592. 22238093 10.1523/JNEUROSCI.2849-11.2012 PMC3287080

[46] George SK, Jiao Y, Bishop CE, Lu B (2011) Mitochondrial peptidase IMMP2L mutation causes early onset of age-associated disorders and impairs adult stem cell self-renewal. Aging cell 10: 584–594. 21332923 10.1111/j.1474-9726.2011.00686.x PMC3111879

[47] Trifunovic A, Wredenberg A, Falkenberg M, Spelbrink JN, Rovio AT, et al (2004) Premature ageing in mice expressing defective mitochondrial DNA polymerase. Nature 429: 417–423. 15164064 10.1038/nature02517

[48] Artal-Sanz M, Tavernarakis N (2009) Prohibitin and mitochondrial biology. Trends in endocrinology and metabolism: TEM 20: 394–401. 19733482 10.1016/j.tem.2009.04.004

[49] Bogenhagen DF, Rousseau D, Burke S (2008) The layered structure of human mitochondrial DNA nucleoids. J Biol Chem 283: 3665–3675. 18063578 10.1074/jbc.M708444200

[50] Birner R, Nebauer R, Schneiter R, Daum G (2003) Synthetic lethal interaction of the mitochondrial phosphatidylethanolamine biosynthetic machinery with the prohibitin complex of *Saccharomyces cerevisiae* . Mol Biol Cell 14: 370–383. 12589040 10.1091/mbc.E02-05-0263 PMC149978

[51] Ballatore C, Lee VM, Trojanowski JQ (2007) Tau-mediated neurodegeneration in Alzheimer's disease and related disorders. Nature reviews Neuroscience 8: 663–672. 17684513 10.1038/nrn2194

[52] Ittner LM, Fath T, Ke YD, Bi M, van Eersel J, et al (2008) Parkinsonism and impaired axonal transport in a mouse model of frontotemporal dementia. Proceedings of the National Academy of Sciences of the United States of America 105: 15997–16002. 18832465 10.1073/pnas.0808084105 PMC2572931

[53] Shahpasand K, Uemura I, Saito T, Asnao T, Hata K, et al (2012) Regulation of Mitochondrial Transport and Inter-Microtubule Spacing by Tau Phosphorylation at the Sites Hyperphosphorylated in Alzheimer's Disease. J Neurosci 32: 2430–2441. 22396417 10.1523/JNEUROSCI.5927-11.2012 PMC6621814

[54] Kopke E, Tung YC, Shaikh S, Alonso AC, Iqbal K, et al (1993) Microtubule-associated protein tau. Abnormal phosphorylation of a non-paired helical filament pool in Alzheimer disease. The Journal of biological chemistry 268: 24374–24384. 8226987

[55] Ferreira A, Lu Q, Orecchio L, Kosik KS (1997) Selective phosphorylation of adult tau isoforms in mature hippocampal neurons exposed to fibrillar A beta. Molecular and cellular neurosciences 9: 220–234. 9245504 10.1006/mcne.1997.0615

[56] Zhu X, Raina AK, Rottkamp CA, Aliev G, Perry G, et al (2001) Activation and redistribution of c-jun N-terminal kinase/stress activated protein kinase in degenerating neurons in Alzheimer's disease. Journal of neurochemistry 76: 435–441. 11208906 10.1046/j.1471-4159.2001.00046.x

[57] Mandelkow EM, Mandelkow E (2012) Biochemistry and cell biology of tau protein in neurofibrillary degeneration. Cold Spring Harbor perspectives in medicine 2: a006247. 22762014 10.1101/cshperspect.a006247 PMC3385935

[58] Martinelli P, La Mattina V, Bernacchia A, Magnoni R, Cerri F, et al (2009) Genetic interaction between the *m*-AAA protease isoenzymes reveals novel roles in cerebellar degeneration. Hum Mol Genet 18: 2001–2013. 19289403 10.1093/hmg/ddp124

[59] Tiveron MC, Hirsch MR, Brunet JF (1996) The expression pattern of the transcription factor Phox2 delineates synaptic pathways of the autonomic nervous system. The Journal of neuroscience: the official journal of the Society for Neuroscience 16: 7649–7660. 8922421 10.1523/JNEUROSCI.16-23-07649.1996 PMC6579082

