# Supplementary figures and images for: Loss of Prohibitin Membrane Scaffolds Impairs Mitochondrial Architecture and Leads to Tau Hyperphosphorylation and Neurodegeneration
Source: PLoS Genet. 2012 Nov 8;8(11):e1003021. doi: 10.1371/journal.pgen.1003021 (PMC3493444; doi:10.1371/journal.pgen.1003021)

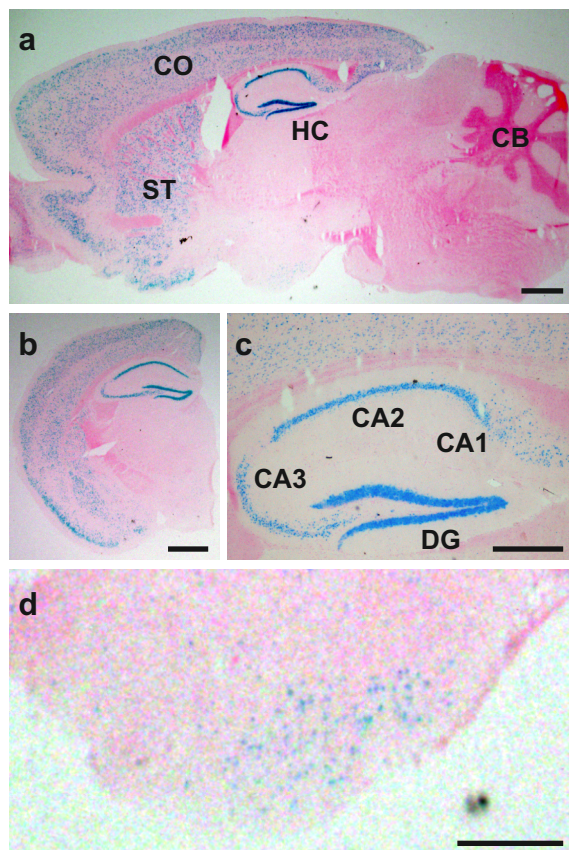

**Figure S1**

Supplement: Figure S1 — Spatially restricted Cre-recombination in mice expressing Cre recombinase under the control of the CaMKIIα promoter. β-galactosidase activity staining of parasagittal (a, d) and coronal sections (b, c) of CaMKIIa-Cre/ROSA26-lacZ reporter brains revealed spatially-restricted Cre recombination in the cortex (CO), the striatum (ST), the hippocampus (HC) and the hypothalamus (d). Maximal recombination efficiency was observed in the hippocampus, in which all neuronal compartments [cornu ammonis (CA), dentate gyrus (DG)] showed strong β-galactosidase staining. CB = cerebellum. Scale bars: 1 mm (a, b); 0,5 mm (c, d). (PDF) [file pgen.1003021.s001.pdf]

**A**

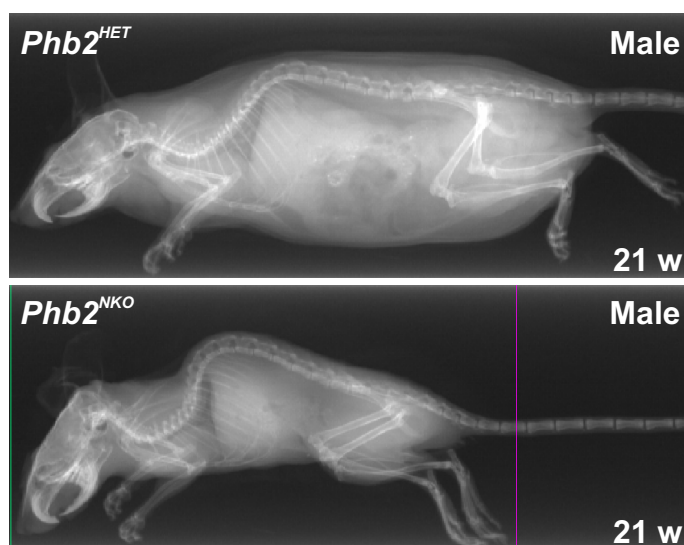

**B**

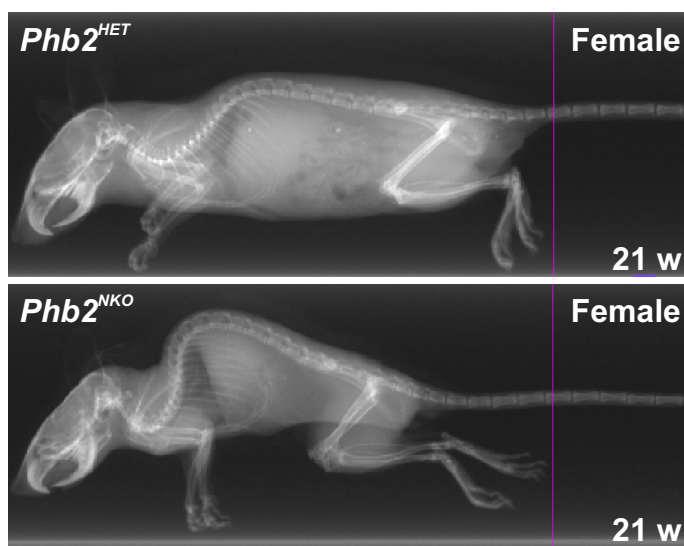

**Figure S2**

Supplement: Figure S2 — Whole-body CT scans of Phb2NKO mice. (A) and (B) Representative Micro-CT scans of 21-week-old (A) male and (B) female Phb2NKO and Phb2HET control mice. Phb2NKO mice displayed a strong curvature of the spinal column (lordokyphosis) and reduction of body size and mass. (PDF) [file pgen.1003021.s002.pdf]

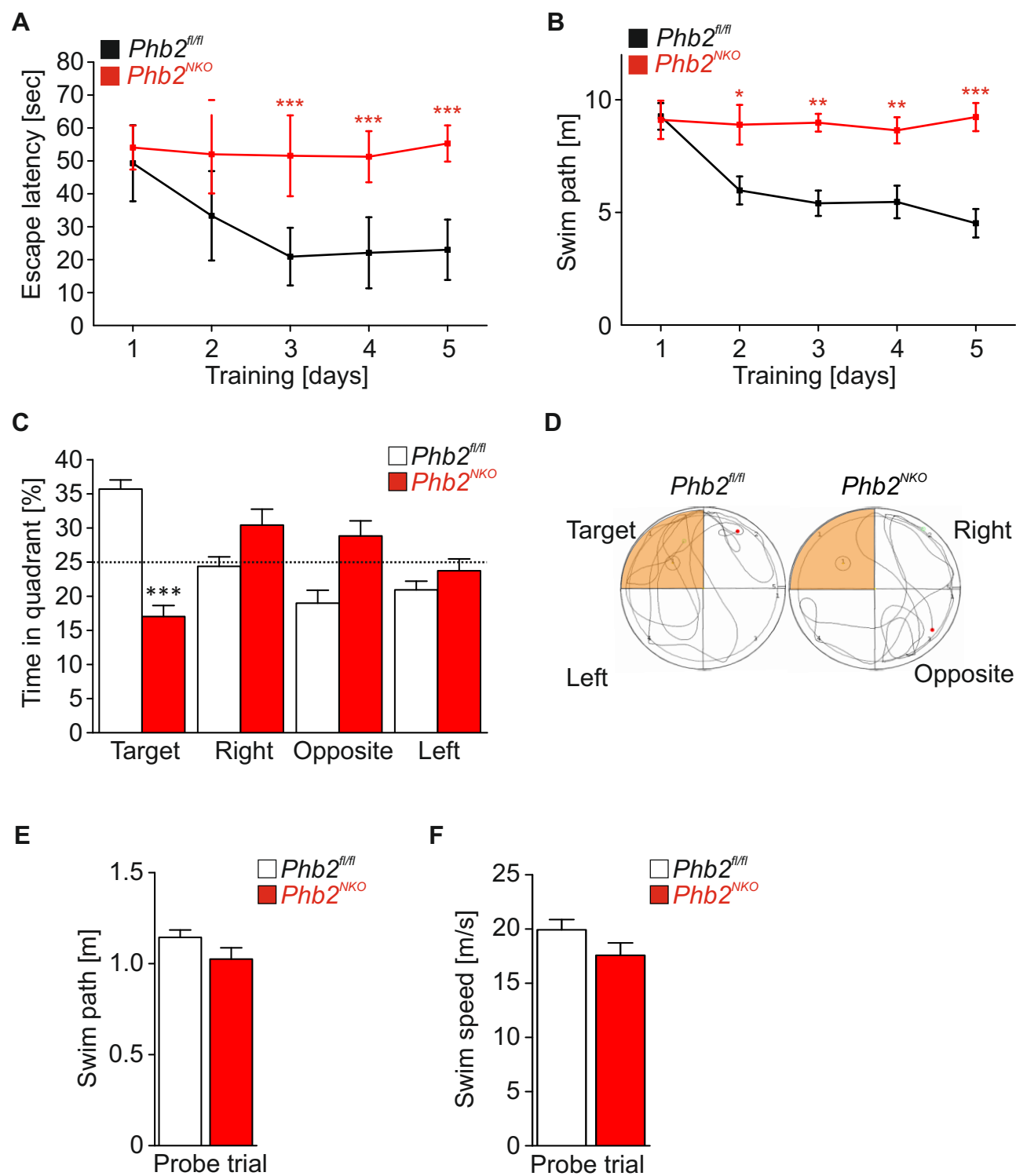

**Figure S3**

Supplement: Figure S3 — Impaired learning and memory abilities of Phb2NKO mice. (A) Escape latencies of 8-week-old Phb2NKO (n = 12) and Phb2fl/fl control mice (n = 13) were examined with the Morris water maze hidden platform paradigm during a 5-day training period. ***P<0.001. Error bars indicate SEM. (B) Swim path comparisons of 8-week-old Phb2NKO (n = 12) and Phb2fl/fl (n = 13) control mice assessed during the training phase in the Morris water maze on five consecutive days. The total distance travelled in four trials per training day is indicated. *P<0.05; **P<0.01; ***P<0.001. Error bars indicate SEM. (C) Swimming times of 8-week-old Phb2NKO (n = 12) and Phb2fl/fl control mice (n = 13) spent in each quadrant in the probe trial on day 5. The dotted line indicates the chance level (25%). ***P<0.001. Error bars indicate SEM. (D) Representative path tracings of 8-week-old Phb2NKO and Phb2fl/fl control mice during the probe trial on day 5. The coloured quadrant indicates the target region after removal of the platform. (E) Swim path comparisons of Phb2NKO mice and Phb2fl/fl controls assessed during the probe trial in the Morris water maze on day 5. Values are expressed as the total distance travelled during 60 s of the probe trial. ***P<0.001. Error bars indicate SEM. (F) Swim velocities of 8-week-old Phb2NKO (n = 12) and Phb2fl/fl (n = 13) control mice assessed during the probe trial in the Morris water maze on day 5. The total distance travelled per 60 sec during the probe trial is indicated. Error bars indicate SEM. (PDF) [file pgen.1003021.s003.pdf]

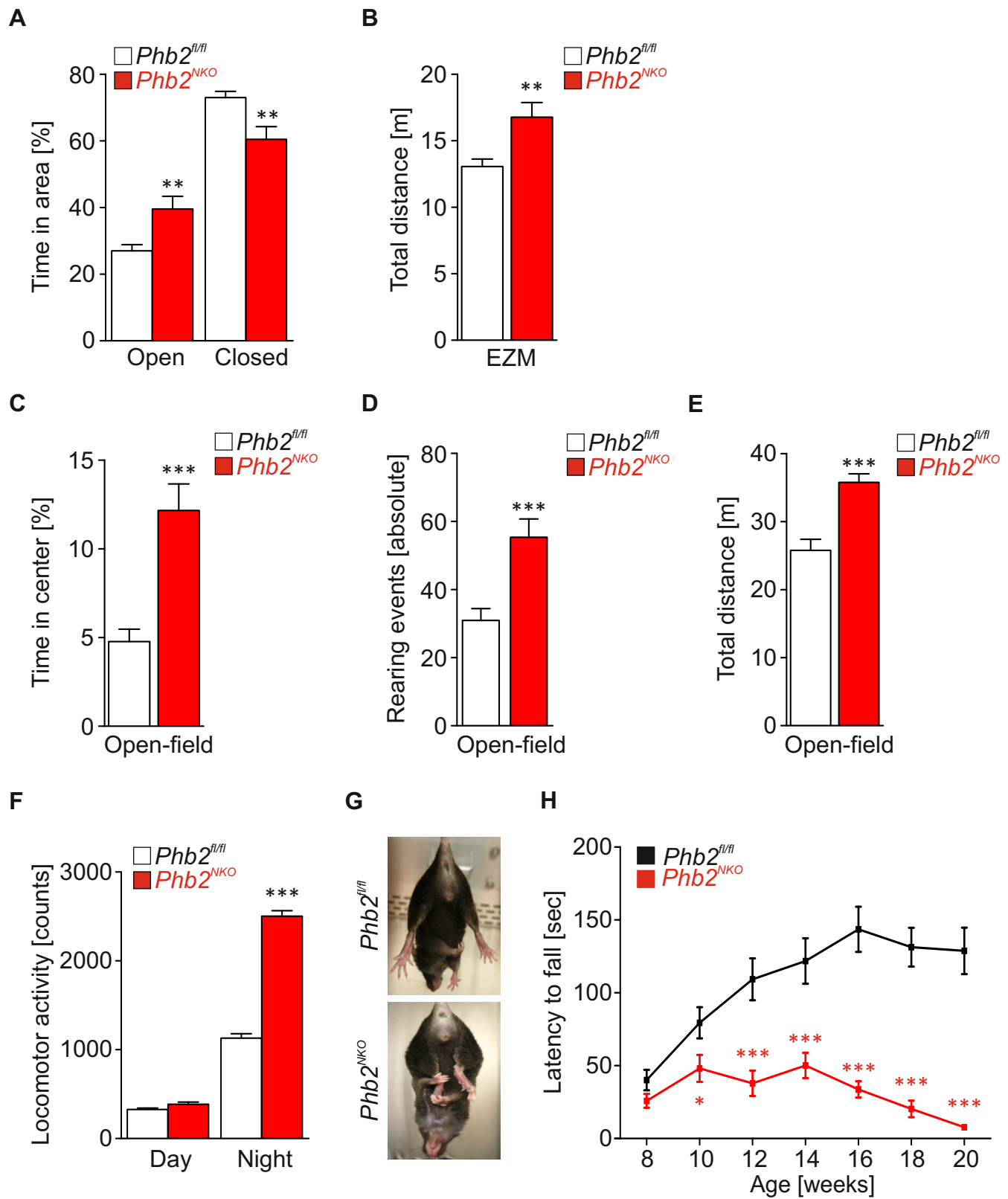

**Figure S4**

Supplement: Figure S4 — Reduced anxiety and loss of motor coordination in Phb2NKO mice. (A) Elevated zero maze analysis of 8-week-old Phb2NKO (n = 12) and Phb2fl/fl control mice (n = 13). Values are expressed as percentage of time spent in either open or closed areas of the maze. **P<0.01. Error bars indicate SEM. (B) Total distance of Phb2NKO (n = 12) and Phb2fl/fl control mice (n = 13) travelled in the elevated zero maze (EZM). **P<0.01. Error bars indicate SEM. (C) Open field test of 8-week-old Phb2NKO (n = 12) and Phb2fl/fl control mice (n = 13). Values are expressed as percentage of time spent in the center of the open field. ***P<0.001. Error bars indicate SEM. (D) Vertical locomotion of 8-week-old Phb2NKO (n = 12) and Phb2fl/fl (n = 13) control mice assessed from total rearing events during a 5-minute test phase in the open field paradigm. ***P<0.001. Error bars indicate SEM. (E) Total distance of Phb2NKO (n = 12) and Phb2fl/fl control mice (n = 13) travelled in the open field. ***P<0.001. Error bars indicate SEM. (F) Locomotor activity of 8-week-old Phb2NKO and Phb2fl/fl control mice during day-night cycle measured in metabolic cages. Data represent total beam break counts during a 12 hour period. n = 4 per group. ***P<0.001. Error bars indicate SEM. (G) Representative photographs of pathological hindlimb clasping reflexes during tail suspension in 18-week-old Phb2NKO mice (lower panel) compared to Phb2fl/fl controls (upper panel). (H) Rotarod performance test of Phb2NKO (n = 12) and Phb2fl/fl control mice (n = 13) examined at the indicated time points. *P<0.05; ***P<0.001. Error bars indicate SEM. (PDF) [file pgen.1003021.s004.pdf]

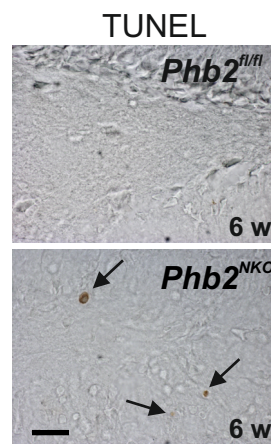

**Figure S5**

Supplement: Figure S5 — Detection of apoptotic DG neurons in Phb2NKO mice. TUNEL staining of DG neurons in 6-week-old Phb2NKO mice is shown (black arrows). Scale bar: 20 µm. (PDF) [file pgen.1003021.s005.pdf]

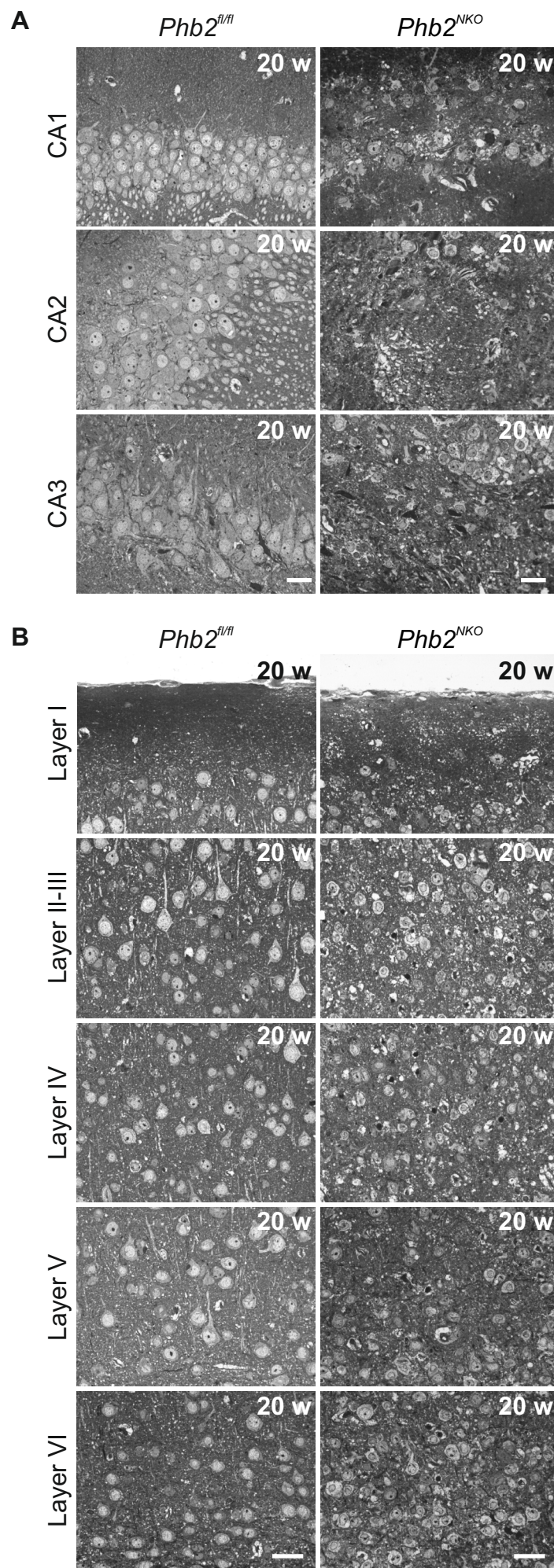

**Figure S6**

Supplement: Figure S6 — Extensive loss of hippocampal and cortical neurons in Phb2NKO mice. (A) Loss of pyramidal neurons in all hippocampal layers of 20-week-old Phb2NKO mice. Coronal semithin sections of the indicated cornu ammonis (CA) areas (CA1, CA2 and CA3) from 20-week-old Phb2NKO and Phb2fl/fl control mice. Scale bars: 20 µm. (B) Late-onset morphological alterations of cerebral cortex neurons in 20-week-old Phb2NKO mice. Coronal semithin sections of cerebral cortex from layers I to VI of 20-week-old Phb2NKO and Phb2fl/fl control mice. Scale bars: 20 µm. (PDF) [file pgen.1003021.s006.pdf]

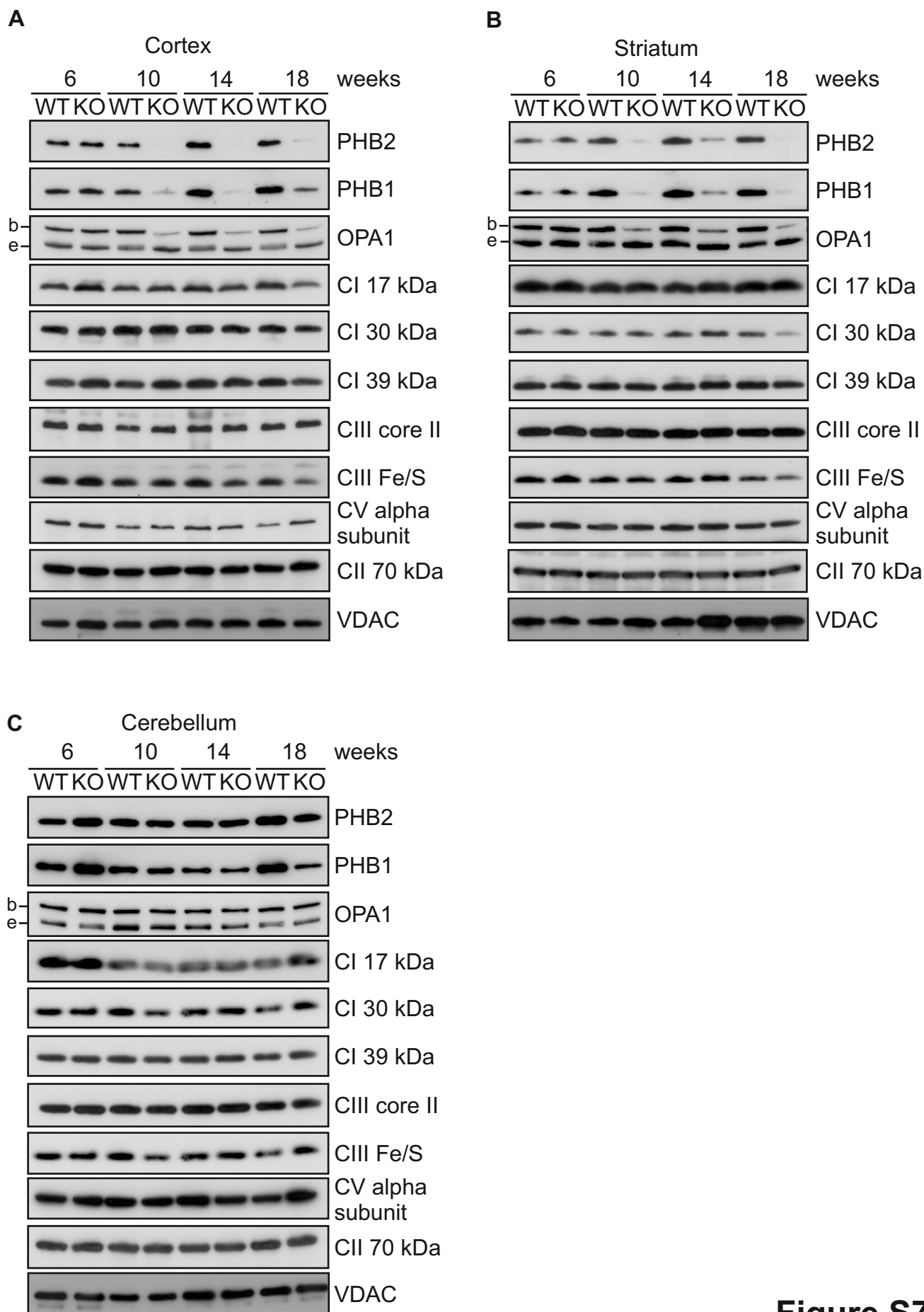

**Figure S7**

Supplement: Figure S7 — Immunoblot analysis of forebrain tissue lysates of Phb2NKO mice. Tissue lysates from cortex, striatum und cerebellum of Phb2NKO (KO) and Phb2 fl/fl (WT) control mice of the indicated age were analyzed by SDS-PAGE and immunoblotting using the indicated antibodies. Antibodies directed against VDAC and the 70 kDa subunit of complex II were used to monitor equal gel loading. b/e: long/short OPA1 isoforms. (PDF) [file pgen.1003021.s007.pdf]

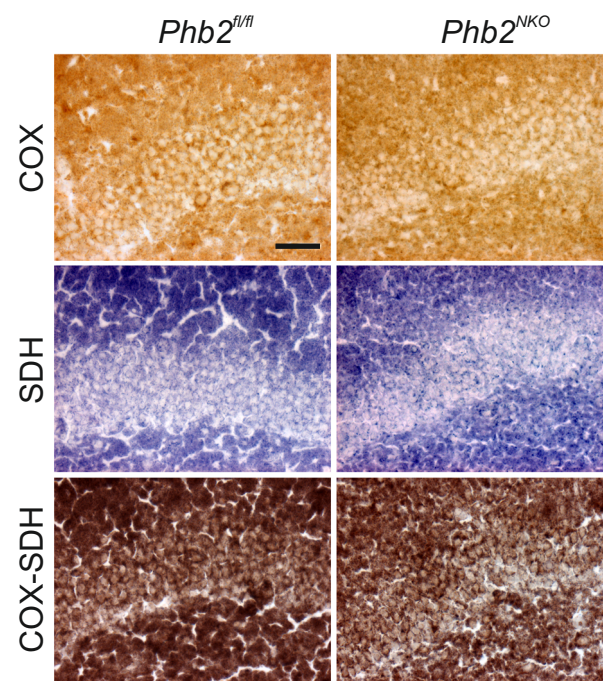

**Figure S8**

Supplement: Figure S8 — COX and SDH activities in DG neurons of 6-week-old Phb2NKO mice. Cross-sections of coronal brain regions from 6-week-old Phb2NKO and Phb2fl/fl control mice were stained for either COX or SDH activities or for both. Representative micrographs are shown. Scale bar: 40 µm. (PDF) [file pgen.1003021.s008.pdf]

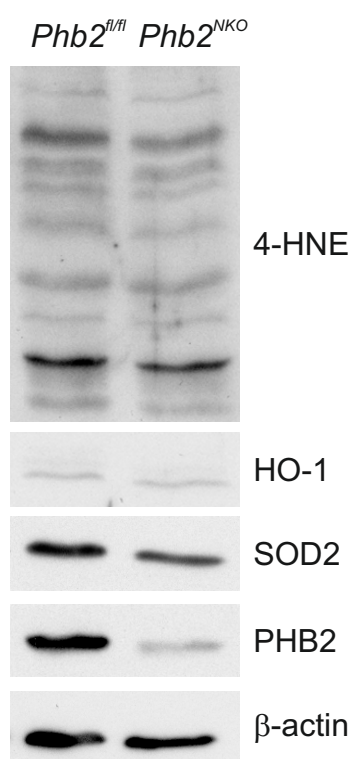

**Figure S9**

Supplement: Figure S9 — Monitoring oxidative damage in Phb2NKO mice. Hippocampal lysates of 14-week-old Phb2NKO and Phb2fl/fl control mice were analyzed by SDS-PAGE and immunoblotting using the indicated antibodies. β-actin was used as a loading control. 4-hydroxynonenal (4-HNE) stainings of coronal sections of the DG of 14-week-old Phb2NKO and Phb2fl/fl control mice did not reveal any signs of lipid oxidation (data not shown). (PDF) [file pgen.1003021.s009.pdf]

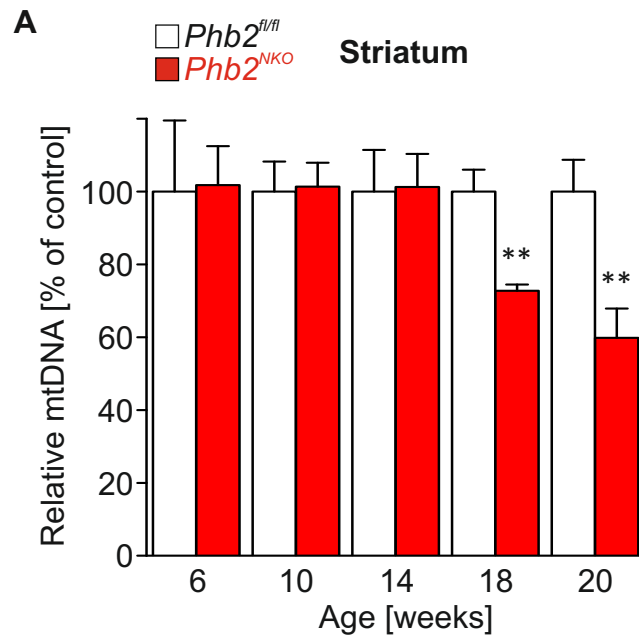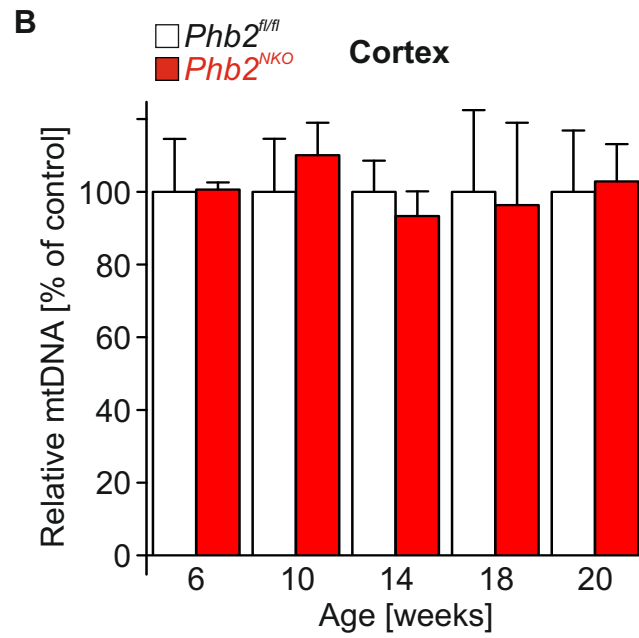

**Figure S10**

Supplement: Figure S10 — Tissue-specific mtDNA loss in PHB2-deficient neurons in vivo. (A) and (B) Relative levels of mtDNA in (A) striatum and (B) cortex of Phb2NKO and Phb2fl/fl control mice. Total DNA was extracted from brain subregions of mice of the indicated age and genotype and analyzed by quantitative real-time PCR analysis using primers specific for mtDNA and nuclear DNA. Data represent average of at least three independent experiments, each sample assayed in quadruples. mtDNA, mitochondrial DNA. Error bars represent SEM. **P<0.01. (PDF) [file pgen.1003021.s010.pdf]
