# Supplementary material for: Loss of Prohibitin Membrane Scaffolds Impairs Mitochondrial Architecture and Leads to Tau Hyperphosphorylation and Neurodegeneration
Source: PLoS Genet. 2012 Nov 8;8(11):e1003021. doi: 10.1371/journal.pgen.1003021 (PMC3493444; doi:10.1371/journal.pgen.1003021)
Supplement: Text S1 — Supporting behavioral studies and supporting methods. (DOCX) [file pgen.1003021.s011.docx]

**Supporting Information**

**Text S1. Supporting information.**

**Behavioral and motor deficits of *Phb2*^NKO^ mice**

To examine whether *Phb2* deficiency impacts on hippocampus-dependent memory formation and learning abilities, we analyzed early-stage 8-week-old *Phb2^NKO^* animals in the Morris water maze (MWZ) paradigm [[1](#_ENREF_1)]. Whereas control animals reduced the escape latency during a 5-day training period for the hidden-platform model, *Phb2^NKO^* mice failed to respond and did not show any improvement throughout the training process (Figure S3A). In addition, swim path comparisons between *Phb2^NKO^* and control mice revealed significant delays in platform location (Figure S3B) pointing to severe deficiencies of *Phb2^NKO^* mice in spatial learning. To further assess hippocampus-dependent memory formation, we removed the platform from the swimming tank in probe trials and determined the time spent in single quadrants. Remarkably, *Phb2^NKO^* mice did not show a preference for the target quadrant while control animals spent significantly more time in this area (Figure S3C, S3D), indicating that loss of prohibitins impairs hippocampus-dependent memory formation. Notably, such learning and memory impairments are likely not due to locomotor dysfunction or lack of motivation since swim performances were comparable between *Phb2^NKO^* mice and littermate controls (Figure S3E, F).

*Phb2* was robustly deleted in both the dentate gyrus (DG) and the cornu ammonis (CA) regions of the hippocampus of *Phb2^NKO^* mice, areas well characterized for regulating anxiety-related behaviors through projections to the amygdala. To assess a role for prohibitins in innate fear control, we analyzed *Phb2^NKO^* mice using elevated zero maze (EZM) and open field (OF) tests. In EZM experiments, *Phb2^NKO^* mice stayed significantly longer in the open- but not in the closed areas of the maze, suggesting a reduced innate-fear response (Figure S4A). An increase in total distance travelled in the EZM indicated hyperactivity of *Phb2^NKO^* mice at this age (Figure S4B). Correspondingly, in OF studies *Phb2^NKO^* mice spend significantly more time in the arena centre compared to control littermates (Figure S4C) and demonstrated an increased exploratory behavior assessed by the quantification of rearing events (Figure S4D). In agreement with the EZM studies, the increase in total distance travelled in the OF indicated hyperactivity of *Phb2^NKO^* mice (Figure S4E) which was independently confirmed by measuring laser beam-brakes of individually housed animals in metabolic cages (Figure S4F). Collectively, the results suggest that ablation of *Phb2* causes anxiolytic behavior of *Phb2^NKO^* mice.

*Phb2^NKO^* mice analyzed at the age of 8 weeks in the described behavioral tests did not reveal abnormalities in body weight, neurological reflexes and energy homeostasis (Figure 1D and data not shown). During aging, however, 18-week-old *Phb2^NKO^* mice exhibited severely impaired motor coordination, including abnormal hindlimb clasping reflexes (Figure S4G), a hallmark feature observed in mouse models of neurodegenerative disease. To monitor motor abilities in more detail, *Phb2^NKO^* mice were tested in the rotarod. Whereas control animals constantly improved rotarod performance over time, *Phb2^NKO^* mice developed profound motor deficits and showed a significantly decreased latency to fall from the rod in a progressive manner (Figure S4H). In conclusion, *Phb2* is required for hippocampus-dependent learning abilities and memory formation, the control of innate fear behavior as well as maintenance of proper motor coordination.

**Supporting Materials and Methods**

**Text S2. Supporting Materials and Methods.**

**Animal care and mouse breedings**

Care of all animals was within institutional animal care committee guidelines and all procedures were approved by local government authorities (Bezirksregierung Köln) and were in accordance with NIH guidelines. Mice were housed in groups of three to five at 22-24°C using a 12 h light/12 h dark cycle with lights on at 6 a.m. Generation of conditional *Phb2* mice has been described [[2](#_ENREF_2)]. *CaMKIIa-Cre* mice (line #159) have been generated as described [[3](#_ENREF_3)]. Mice were genotyped by PCR using genomic DNA isolated from tail biopsies.

**Isolation and cultivation of primary hippocampal neurons**

Primary hippocampal neurons were isolated from E18.5 conditional *Phb2^fl/fl^* or *Phb2^fl/wt^* embryos according to standard procedures [[4](#_ENREF_4)]. Embryos were collected from time-mated pregnant females. Specifically, *Phb2^fl/fl^* embryos were derived from intercrosses of male and female *Phb2^fl/fl^* mice, whereas *Phb2^fl/wt^* embryos were obtained from C57BL/6 females bred with male *Phb2^fl/fl^* mice. Hippocampal neurons were plated on poly-L-lysine-coated glass coverslips and cultured in Neurobasal medium (Invitrogen) supplemented with B27 (Invitrogen), 0.5 mM L-glutamine (PAA), 100 U/ml penicillin (PAA) and 100 µg/ml streptomycin (PAA). After 24 h the coverslips were transferred, face down, to dishes containing monolayers of cortical glial cells [[5](#_ENREF_5)]. For long-term growth, neurons were cultured in Neurobasal-A medium (Invitrogen) supplemented with B27 (Invitrogen), 0.5 mM L-glutamine (PAA), 100 U/ml penicillin (PAA), 100 µg/ml streptomycin (PAA), 25 µM 2-mercaptoethanol and 10 ng/ml bFGF (Invitrogen). Cells were cultured at 37°C in an atmosphere of 5% CO_2_ and 90% humidity.

**Generation and infection of lentiviral particles**

Lentiviral particles were generated with the ViraPower™ Lentiviral Expression System according to the manufacturer´s instructions (Invitrogen, Karlsruhe, Germany). cDNAs for Su9-EGFP [[6](#_ENREF_6)]and NLS-Cre [[7](#_ENREF_7)]were PCR-amplified and cloned into the pENTR/siH1/mDD-stuffer plasmid [[8](#_ENREF_8)]. Lentiviral pLenti6 vectors expressing the CMV promoter-driven transgenes were generated by LR recombination (Invitrogen, Karlsruhe, Germany). Lentiviral particles were produced in HEK293FT cells, collected after 48 h, filtered and concentrated by ultracentrifugation. Lentiviral titers were determined in CV1-5B reporter cell lines [[9](#_ENREF_9)]. Primary hippocampal neurons were transduced at DIV=7 with concentrated lentiviral supernatants in Neurobasal medium supplemented with N2 (Invitrogen), 0.5 mM L-glutamine (PAA), 100 U/ml penicillin (PAA) and 100 µg/ml streptomycin (PAA) at an MOI of 100 for 12 h at 37°C and processed for immunocytochemistry.

**Immunocytochemistry**

Cells were rinsed with ice-cold PBS, fixed with 4% paraformaldehyde in PBS for 15 min and permeabilized with 0.15% Triton X-100 in phosphate-buffered saline (PBS) for 20 min. Coverslips were incubated in blocking solution (3% BSA in 0.01% PBS-Triton X-100) for 30 min at room temperature and immunostained with primary antibodies (anti-rabbit EGFP antibody (ab6556, 1:1000, Abcam); anti-mouse monoclonal βIII-tubulin (1:1000, Sigma, Germany) diluted in blocking solution overnight at 4°C. After rinsing with 0.01% PBS-Triton X-100, cells were incubated with appropriate Alexa-conjugated secondary antibodies (goat anti-rabbit Alexa-488 and goat anti-mouse Alexa-546, 1:1000, Invitrogen) diluted in blocking solution for 1 h at at 4°C in the dark. Cells were stained with DAPI (Roche, Germany) for 5 min, rinsed extensively with PBS and mounted with ProLong Gold Antifade reagent (Invitrogen). High resolution images were acquired using a DeltaVision microscope system (Applied Precision).

**Quantitative real-time-PCR of mitochondrial DNA (mtDNA)**

Quantification of mtDNA was performed as previously described [[10](#_ENREF_10)]. Total DNA was isolated from brain regions including the hippocampus, cerebral cortex, striatum and the cerebellum by standard procedures. Real-time amplification was performed using SYBR^®^ Green PCR Master Mix (Applied Biosystem, Foster City, CA, USA) in an ABI PRISM 7000 Sequence Detection System according to the manufacturer´s instructions. mtDNA was detected with primers specific for the murine *COI* gene [[11](#_ENREF_11)]. Amplification of the nuclear-encoded murine *RNaseP* was used as nuclear gene standard reference. Each PCR reaction was performed in triplicate. Relative abundance of mtDNA was determined using a comparative method (2^-ΔΔCT^) according to the ABI Relative Quantification Method.

**Immunoblotting**

Brain tissues were dissected and homogenized using a Polytron homogenizer (IKA Werke, Germany) in ice-cold lysis buffer (50 mM HEPES/KOH pH 7.4, 10 mM EDTA, 50 mM NaCl, 100 mM NaF, 1% Triton X-100, 0.1% SDS supplemented with 20 µg/ml aprotinin, 2 mM benzamidine, 10 mM sodium-orthovanadate, 1 mM PMSF and complete protease inhibitor cocktail mix (Roche)). Protein concentrations were determined with a standard Bradford protein assay (BioRad). 50-200 µg of total protein were separated on SDS-PAGE, transferred to nitrocellulose membranes (VWR, Germany) and subjected to immunoblotting with the following antibodies: rabbit anti-BAP37 (#6118, BioLegend, 1:500), rabbit anti-prohibitin (#6031, BioLegend, 1:500), mouse anti-OPA1 (BD Biosciences, 1:500), mouse anti-PHF-TAU (#MN1020, clone #AT8, Thermo Scientific, 1:500), rabbit anti-SAPK/JNK (#9258, Cell Signaling, 1:1000), rabbit anti-phospho-SAPK/JNK (T183/Y185) (#9251, Cell Signaling, 1:1000), rabbit anti-p44/42 (Erk1/2) (#9102, Cell Signaling, 1:1000), rabbit anti-phospho-p44/42 MAPK (Erk1/2) (T202, Y204) (#9101, Cell Signaling, 1:1000), rabbit anti-AKT (pan) (#9272, Cell Signaling, 1:1000), rabbit anti-phospho-AKT (S473) (#9271, Cell Signaling, 1:1000), rabbit anti-GSK3β (#9315, Cell Signaling, 1:1000), rabbit anti-phospho- GSK3β (S9) (#9323, Cell Signaling, 1:1000), rabbit anti-p35/p25 (#2680, Cell Signaling, 1:1000), and mouse anti-β-actin (#A5541, Sigma, 1:5000).

Mitochondrial respiratory chains subunits were determined via immunoblotting using the following antibodies: mouse anti-complex I, 17 kDa (NDUFB6, clone 21C11, Molecular Probes, 1:1000), mouse anti-complex I, 30 kDa (NDUFS3, clone 3F9, Molecular Probes, 1:1000), mouse anti-complex I, 39 kDa (NDUFA9, clone 20C11, Molecular Probes, 1:1000), mouse anti-complex II, 70 kDa (SDHA, clone 2E3, Molecular Probes, 1:1000), mouse anti-complex III, Fe/S (UQCRFS1, clone 5A5, Molecular Probes, 1:1000), mouse anti-complex III, Core II (UQCRC2, clone 13G12, Molecular Probes, 1:1000), mouse anti-complex IV, subunit I (MTCO1, clone 1D6, Molecular Probes, 1:1000), mouse anti-complex IV, subunit Vb (COX5B, clone 16H12, Molecular Probes, 1:1000), mouse anti-complex V, subunit α (ATP5A1, clone 7H10, Molecular Probes, 1:1000).

**Assessing mitochondrial respiratory function**

Oxygraphic measurements of isolated neuronal mitochondria using a specific substrate inhibitor titration protocol were performed with the Oxygraph-2k (Oroboros Instruments, Austria) as previously described [[12](#_ENREF_12)]. Mitochondria were isolated from freshly dissected murine brain tissues. Briefly, tissues were cut into small pieces and homogenized in ice-cold isolation buffer (220 mM mannitol, 70 mM sucrose, 20 mM HEPES/KOH pH 7.4, 2 mM EGTA and 0.1% BSA) with 10 strokes in a motor-driven homogenizer (B. Braun, Germany). The crude homogenate was centrifuged at 1.000 *g* for 10 min at 4°C. The supernatant was centrifuged at 8.000 *g* for 10 min at 4°C to pellet mitochondria. Mitochondrial protein concentrations were determined with a standard Bradford protein assay (BioRad). The two chambers of the Oxygraph-2k were washed with water and calibrated with 2.3 ml brain medium (10 mM potassium dihydrogenphosphate, 60 mM potassium chloride, 60 mM Tris/HCl pH 7.4, 110 mM mannitol, 0.5 mM EDTA) for each chamber. After calibration 300 μg of the mitochondrial fraction, 5 mM MgCl_2_, 10 mM pyruvate and 500 μM malate were added into both chambers. The following chemicals (Sigma, Germany) were used at the indicated concentrations: 2 mM ADP, 10 mM glutamate, 10 mM malate, 1 mM amytal (chamber A) or 0.5 μM rotenone (chamber B), 10 mM succinate, 10 mM malonate, 1 mM TMPD + ascorbate, 10 μM cytochrome *c*, 5 mM potassium cyanide. Measurements were carried out at 30°C. Chambers were washed between measurements to remove mitochondria and chemicals.

**Blue native polyacrylamide gel electrophoresis (BN-PAGE)**

BN-PAGE of native mitochondrial respiratory chain complexes was performed as described [[10](#_ENREF_10)]. Crude neuronal mitochondria (100 μg) isolated from murine brain regions were solubilized for 30 min at a concentration of 2.5 mg/ml in solubilisation buffer (50 mM NaCl, 5 mM 6-aminohexanoic acid, 50 mM imidazole/HCl, pH 7.0, 10% glycerol, 50 mM potassium phosphate) supplemented with detergent (8 g/g digitonin or 0.8% DDM). Solubilized material was centrifuged for 30 min at 20.000 *g*, Coomassie Brilliant Blue G-250 was added and samples were loaded on native gels composed of a gradient separation gel (3-13% polyacrylamide) and a 3% stacking gel. Gels were either transferred onto a PVDF membrane by semi-dry electroblotting for 3 h at 400 mA or stained with colloidal Coomassie staining solution. Thyroglobulin (669 kDa) and apoferritin (443 kDa) were used as markers.

**Behavioral analyses**

Behavioral studies were conducted with cohorts of 8-week-old *Phb2^NKO^* (n=12) and *Phb2^fl/fl^* (n=13) control mice in the following order: Morris mater maze, elevated zero maze, rotarod and open field. Separate cohorts of mice were used for indirect calorimetry in metabolic chambers (n=4) and for microCT analysis (n=9). Behavioral data were obtained in blind-coded experiments.

**Morris water maze**

Spatial learning and memory abilities were tested with the hidden platform test of the Morris water maze paradigm [[1](#_ENREF_1)]. A white plastic tank with a diameter of 120 cm was filled with room temperature water. A platform (10 cm diameter) was submerged at a constant location 1.4 cm below the water surface in the centre of the target quadrant during the five day training phase. External cues were provided as spatial references outside of the tank. For each training session, the mice were placed into the maze from four appointed positions of the tank. Mice were allowed to find the platform within 60 s. If the mice did not find the platform within 60 s, they were placed on it. Mice were allowed to remain on the platform for 15 s. Four consecutive training trials were given every day; the latency for each trial was recorded for analysis. During the memory test (probe trial), the platform was removed from the tank, and the mice were allowed to swim in the maze for 60 s. Swim paths and velocities of the mice were recorded by a video camera and analyzed by Videomot 2 software V5.76 (TSE Systems GmbH, Bad Homburg, Germany).

**Elevated zero maze**

Anxiety-related behaviors were assessed with the elevated zero maze test [[13](#_ENREF_13)]. A 5.5 cm wide circular runway with an outer diameter of 47 cm was placed 50 cm above the ground. Two opposing quadrants were enclosed by 11 cm high walls (closed sectors) whereas the remaining quadrants did not contain walls (open sectors). Mice were placed on the brightly illuminated (460 to 520 lux) open part of the apparatus (closed part 360 to 390 lux), and their behavior was recorded and analyzed for 5 minutes using an automated video tracking system (VideoMot 2 V5.76, TSE Systems GmbH, Bad Homburg, Germany). Time spent in the open/closed sections was evaluated.

**Open field test**

Anxiety and locomotor activity were examined in the novel open field test [[14](#_ENREF_14)]. Mice were recorded and analyzed over a 5 minute period using an automated video tracking system (VideoMot 2, V5.76), in an open field box made of grey plastic with 50 cm x 50 cm surface area and 30 cm-high walls (both TSE Systems GmbH, Bad Homburg, Germany). Tracing paths of the mice were recorded and time spent in the central part (25 cm x 25 cm) versus time spent at the border was evaluated.

**Rotarod**

Motor coordination of mice was analyzed in the rotarod performance test [[15](#_ENREF_15)]. The apparatus (TSE Systems, Bad Homburg, Germany) was set to accelerate from 4 to 40 rpm in 300 sec and one test session consisted of three trials separated by 15 min inter-trial intervals. Latency to fall from the accelerating rotarod was assessed over a period of 13 consecutive weeks with one test session per week at the age of 8 to 20 weeks.

**Indirect calorimetry and physical activity**

Metabolic rate measurements were performed in an open circuit calorimetry system (PhenoMaster, TSE Systems GmbH, Bad Homburg, Germany). Mice were placed at room temperature (22°C-24°C) in regular type II cages with sealed lids of the PhenoMaster open circuit calorimetry. Mice were allowed to adapt to the chambers for at least 24 hours. Food and water were provided *ad libitum* in the appropriate devices. The system was further equipped with an infrared light-beam frame to determine the home cage activity (ActiMot2, TSE Systems GmbH, Bad Homburg, Germany). Parameters of indirect calorimetry were measured for the following 48 hours. Presented data are average values obtained in these recordings.

**Computed tomography analysis**

For morphometry and body composition measurements a LaTheta LCT-100 (Aloka Co. LTD., Tokyo, Japan) micro computed tomography scanner was used. The X-ray source tube voltage was set at 50 kV with a constant 1 mA current. A holder with inner diameter of 48 mm was used, resulting in pixel resolutions of 100 μm. Pitch was 0.5 mm and scan speed 4.5 s/slice. LaTheta software V2.10 estimates the volumes of adipose tissue, bone, air, and the remainder using differences in X-ray density. The thresholds for fat mass and lean mass were set at -500 to -120 HU and -120 to 350 HU, respectively. A ketamin/xylazine anesthesia has been used to immobilize the animals.

**Statistical analysis**

Data sets were analyzed for statistical significance using a two-tailed unpaired Student´s t-test. All data represent mean values ± SEM. **P*<0.05; ***P*<0.01; ****P*<0.001 versus control.

**Supporting references**

1. Morris RG, Garrud P, Rawlins JN, O'Keefe J (1982) Place navigation impaired in rats with hippocampal lesions. Nature 297: 681-683.

2. Merkwirth C, Dargazanli S, Tatsuta T, Geimer S, Lower B, et al. (2008) Prohibitins control cell proliferation and apoptosis by regulating OPA1-dependent cristae morphogenesis in mitochondria. Genes Dev 22: 476-488.

3. Minichiello L, Korte M, Wolfer D, Kuhn R, Unsicker K, et al. (1999) Essential role for TrkB receptors in hippocampus-mediated learning. Neuron 24: 401-414.

4. Fath T, Ke YD, Gunning P, Gotz J, Ittner LM (2009) Primary support cultures of hippocampal and substantia nigra neurons. Nature protocols 4: 78-85.

5. Kaech S, Banker G (2006) Culturing hippocampal neurons. Nature protocols 1: 2406-2415.

6. Chen H, Detmer SA, Ewald AJ, Griffin EE, Fraser SE, et al. (2003) Mitofusins Mfn1 and Mfn2 coordinately regulate mitochondrial fusion and are essential for embryonic development. J Cell Biol 160: 189-200.

7. Gu H, Zou YR, Rajewsky K (1993) Independent control of immunoglobulin switch recombination at individual switch regions evidenced through Cre-loxP-mediated gene targeting. Cell 73: 1155-1164.

8. Pongratz C, Yazdanpanah B, Kashkar H, Lehmann MJ, Krausslich HG, et al. (2010) Selection of potent non-toxic inhibitory sequences from a randomized HIV-1 specific lentiviral short hairpin RNA library. PloS one 5: e13172.

9. Kellendonk C, Tronche F, Monaghan AP, Angrand PO, Stewart F, et al. (1996) Regulation of Cre recombinase activity by the synthetic steroid RU 486. Nucleic acids research 24: 1404-1411.

10. Martinelli P, La Mattina V, Bernacchia A, Magnoni R, Cerri F, et al. (2009) Genetic interaction between the *m*-AAA protease isoenzymes reveals novel roles in cerebellar degeneration. Hum Mol Genet 18: 2001-2013.

11. Spinazzola A, Viscomi C, Fernandez-Vizarra E, Carrara F, D'Adamo P, et al. (2006) MPV17 encodes an inner mitochondrial membrane protein and is mutated in infantile hepatic mitochondrial DNA depletion. Nature genetics 38: 570-575.

12. Kuznetsov AV, Veksler V, Gellerich FN, Saks V, Margreiter R, et al. (2008) Analysis of mitochondrial function in situ in permeabilized muscle fibers, tissues and cells. Nature protocols 3: 965-976.

13. Shepherd JK, Grewal SS, Fletcher A, Bill DJ, Dourish CT (1994) Behavioural and pharmacological characterisation of the elevated "zero-maze" as an animal model of anxiety. Psychopharmacology 116: 56-64.

14. Crawley JN (2007) Mouse behavioral assays relevant to the symptoms of autism. Brain pathology 17: 448-459.

15. Jones BJ, Roberts DJ (1968) The quantitative measurement of motor inco-ordination in naive mice using an acelerating rotarod. The Journal of pharmacy and pharmacology 20: 302-304.
